# Supplementary material for: Evaporate-casting of curvature gradient graphene superstructures for ultra-high strength structural materials
Source: Nat Commun. 2024 Jul 14;15:5917. doi: 10.1038/s41467-024-50191-6 (PMC11247093; doi:10.1038/s41467-024-50191-6)
Supplement: Supplementary file 1 — Supplementary Information [file 41467_2024_50191_MOESM1_ESM.pdf]

Supplementary Information for

Evaporate-casting of Curvature Gradient Graphene Superstructures for Ultra-high Strength Structural Materials

Bing Lu[1,2], Li Yu[3], Yajie Hu[1], Ying Wang[1], Fei Zhao[2], Yang Zhao[2], Feng Liu[3*], Huhu Cheng[1], Liangti Qu[1*]*

This supplementary information includes:

Supplementary text

Supplementary Figures 1-30

Supplementary Table 1

Supplementary text

Theoretical model

Due to the gradient of curvature in the graphene wall within the sample, graphene hydrogel undergoes bending deformation upon contraction. The following configurations are analyzed to investigate the relationship between the distribution morphology of graphene and bending deformation. The graphene walls are composed of multiple sets of "cylinders" with different curvatures. The axis of each cylinder males an angle of θ with the normal of the sample plane. In this part, the relationship between ε_r and ρ is set as $\varepsilon_r = \varepsilon_0 + b\rho^2$. For the cylinder samples, the curvature radius ρ is equal to r, where r is the distance from graphene walls to the cylinder's axis. Thus, the radial strain of the graphene wall can be written as $\varepsilon_r = \varepsilon_0 + br^2$. In cylindrical coordinates, the strain compatibility equation for axis-symmetric deformation can be expressed as follows:

$$-\frac{\partial^2 \varepsilon_{\varphi\varphi}}{\partial r^2} + \frac{1}{r}\frac{\partial \varepsilon_r}{\partial r} - \frac{2}{r}\frac{\partial \varepsilon_{\varphi\varphi}}{\partial r} = 0 \tag{3.1}$$

Thus, the tangential strain can be written as $\varepsilon_{\varphi\varphi}=\varepsilon_0+br^2/3$. In the cylindrical coordinate system $\{r, \varphi, z^*\}$ of the graphene wall, the strain tensor ε can be expressed as follows:

$$\boldsymbol{\varepsilon} = \begin{bmatrix} \varepsilon_{rr} & 0 & 0 \\ 0 & \varepsilon_{\varphi\varphi} & 0 \\ 0 & 0 & 0 \end{bmatrix} \tag{3.2}$$

The transformation matrix between the cylindrical coordinate system of the graphene wall and rectangular coordinate $\{x, y, z\}$ of the sample, as shown in Supplementary Fig.15, is given by,

$$\mathrm{C} = \begin{bmatrix} \cos\theta\cos\varphi & \sin\varphi & -\sin\theta\cos\varphi \\ -\cos\theta\sin\varphi & \cos\varphi & \sin\theta\sin\varphi \\ \sin\theta & 0 & \cos\theta \end{bmatrix} \tag{3.3}$$

The strain tensor components in the rectangular coordinate system $\{x, y, z\}$ can be expressed as a result of the coordinate transformation $\varepsilon' = C\varepsilon C^{\mathrm{T}}$, where 'T' represents the transpose.

$$\begin{aligned} \varepsilon_{xx} &= \varepsilon_{rr}\cos^2\theta\cos^2\varphi + \varepsilon_{\varphi\varphi}\cos^2\theta\sin^2\varphi \\ \varepsilon_{yy} &= \varepsilon_{rr}\sin^2\varphi + \varepsilon_{\varphi\varphi}\cos^2\varphi \\ \varepsilon_{zz} &= \varepsilon_{rr}\sin^2\theta\cos^2\varphi + \varepsilon_{\varphi\varphi}\sin^2\theta\sin^2\varphi \end{aligned} \tag{3.4}$$

Based on the results above, we can analyze the bending behavior of cylindrical graphene hydrogels. Due to the complex overall deformation, the deformation of the sample is analyzed in three independent planes (*x-y*, *x-z*, *y-z*).

Firstly, an analysis is conducted on the narrow strip at $y = 0$ (that is, $d = 0$ mm in experiment) within the sample, which exhibits the most significant deformation in the *x-z* plane. Here, the graphene wall is perpendicular to the *x-z* plane, and deformations within the *x-y* plane can be neglected, as shown in the red line in Supplementary Fig. 16a. As the graphene hydrogel dries, the sample undergoes strains pointing toward the axis of the cylinders, as shown in Supplementary Fig.16b. The configuration can be regarded as composed of multiple bands parallel to the z^* axis. With each of these bands, the strain is uniform, related to the curvature radius of the graphene wall at that location. Hence, it is straightforward to obtain the displacement of the central slice in the sample.

$$u_r = \int_0^x \varepsilon_{rr} dx \cos\theta = x\varepsilon_0 \cos\theta + \frac{1}{3} b x^3 \left(\cos\theta\right)^3 \tag{3.5}$$

After deformation, the displacement of various points within the sample is as follows:

$$\begin{cases} u_x = u_r \cos\theta \\ u_z = -u_r \sin\theta \end{cases} \tag{3.6}$$

The shapes of the deformed samples ($\varepsilon_0 = -0.9$, $b = 0.9$) are shown in Supplementary Fig.16d. Combine these theoretical results with the experiment part, when $d = 0$, the deformation of *cg-G* slice is determined by its θ value and size (x value). Furthermore, for *cg-G* slices with same size and profiles, their deformation can be fully determined by d and θ values.

In this part, the deformation within the *x-y* plane will proceed with the analysis, as shown in Supplementary Fig.17a and 17b. The deformation is assumed to approximately satisfy the assumption of plane sections, allowing us to utilize beam bending theory for analysis. Due to the curvature gradient within the sample, deformation during contraction is not uniform. While undergoing axial deformation along the *y*-axis, there is also bending deformation resulting from uneven deformation of the upper and lower surfaces. The relationship between the curvature of bending and the deformation is given by

$$\frac{\left(\rho_{x-y} + t/2\right) d\varphi - \left(\rho_{x-y} - t/2\right) d\varphi}{\rho_{x-y} d\varphi} = \frac{\left(1+\varepsilon_{yy-A}\right) dx - \left(1+\varepsilon_{yy-B}\right) dx}{dx} \tag{3.7}$$

where $\varepsilon_{yy\text{-}A}$ and $\varepsilon_{yy\text{-}B}$ respectively denote the projections of strains at points A and B on the upper and lower surfaces of the sample along the y-axis. The components of strain along the y-axis are,

$$\begin{aligned}\varepsilon_{yy-A} &= \varepsilon_{yy}\left(x_0 - \frac{t}{2}, y, 0\right) = \varepsilon_0 + by^2 + \frac{1}{3}b\left(x_0 - \frac{t}{2}\right)^2 \cos^2\theta \\ \varepsilon_{yy-B} &= \varepsilon_{yy}\left(x_0 + \frac{t}{2}, y, 0\right) = \varepsilon_0 + by^2 + \frac{1}{3}b\left(x_0 + \frac{t}{2}\right)^2 \cos^2\theta\end{aligned} \tag{3.8}$$

Thus, Eq. (3.7) can be written as,

$$\rho_{x-y} = \frac{t}{\varepsilon_{yy}\left(x_0 - \frac{t}{2}, y, 0\right) - \varepsilon_{yy}\left(x_0 + \frac{t}{2}, y, 0\right)} = \frac{1}{-\frac{2}{3}bx_0\cos^2\theta} \tag{3.9}$$

where x_0 is the distance between *cg-G* slice and axis, equal to the d value in the experiment section. When the rotation angle is small, the relationship between the displacement u_x and the bending curvature of the sample is given by,

$$\frac{1}{\rho} = \frac{d^2u_x}{dy^2} = -\frac{2}{3}bx_0\cos^2\theta \tag{3.10}$$

Finally, displacement u_x can be obtained,

$$u_x = -\frac{1}{3}bx_0y^2\cos^2\theta \tag{3.11}$$

The axis deformation of the sample is easy to obtain,

$$u_y = \int_o^y \varepsilon_{yy}dy = \left(\varepsilon_0 + \frac{1}{3}bx_0^2\cos^2\theta\right)y + \frac{1}{3}by^3 \tag{3.12}$$

From Eq. (3.11), it can be observed that as the slice moves away from the center of the cylinder, the sample exhibits greater bending deformation in the x-y plane. When the graphene wall is perpendicular to the sample, i.e., $\theta = 0°$, the bending deformation in the x-y plane is maximized. As θ increases, the bending deformation gradually decreases until it reaches zero.

The curvature radius of bending deformation in the y-z plane can be determined by the same method.

$$\rho_{y-z} = \frac{t}{\varepsilon_{yy}(x_0, y, -\frac{t}{2}) - \varepsilon_{yy}(x_0, y, \frac{t}{2})} = \frac{1}{\frac{2}{3}bx_0\cos\theta\sin\theta} \tag{3.13}$$

The z-direction displacement, denoted as u_z, resulting from the bending deformation in the y-z plane can be expressed as follows:

$$u_z = \frac{1}{3} b x_0 y^2 \cos\theta \sin\theta \tag{3.14}$$

From the above equation, it is evident that the bending deformation in the *y-z* plane reaches its maximum value at $\theta = 45°$. Further combine these theoretical results with the experiment part. For the *cg-G* slice with *d* value not equal to zero ($x_0 \neq 0$), the deformation of *cg-G* slice is determined by its *d* and θ values, and size (*y* value). For the *cg-G* slices with same size and profiles, their deformation can be fully determined by *d* and θ values.

In the above analysis, the deformation of the entire sample on three different planes has been analyzed separately, assuming a deformation field directly and neglecting the internal forces within the material during the deformation process. While this approach may not be absolutely accurate, it greatly simplifies calculations, leading to simple and physically clear formulas that are sufficient for qualitatively describing the deformation characteristics of the sample in experimental settings.

Supplementary Figures

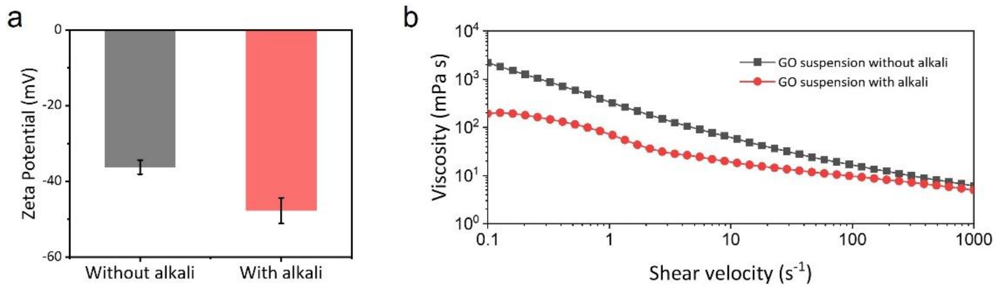

Supplementary Figure 1. The impact of alkali addition on GO sheets. a, Zeta potentials (n = 3, error bar: standard deviation), and **b**, apparent viscosities of GO suspension (3.5 mg mL^{-1}) with/without ammonia hydroxide.

To explore the impact of alkali addition on GO sheets, we characterized the surface charge and rheological behaviors of GO suspension before and after treatment with ammonia. Upon the addition of ammonia hydroxide (the volume ratio of GO aqueous dispersion to 32% ammonium hydroxide is 60:1), the Zeta potential of the GO dispersion underwent a notable shift, declining from an initial -36.2 mV to approximately -47.7 mV (Fig. S1a). This suggests that ammonia can effectively stimulate the ionization of oxygen-containing functional groups (like carboxyl groups) on the surface of GO, thereby intensifying the electrostatic repulsion between GO sheets. Consequently, this enhancement in electrostatic repulsion dynamically promotes the formation of a liquid crystal phase. Specifically, the robust electrostatic repulsion between GO sheets significantly enhances the fluidity of the system (Fig. S1b), facilitating the adjustment of GO sheets' orientation in response to external disturbance. This adjustment enables them to migrate to the position of minimal free energy, ultimately resulting in the formation of a long-range ordered liquid crystal phase.

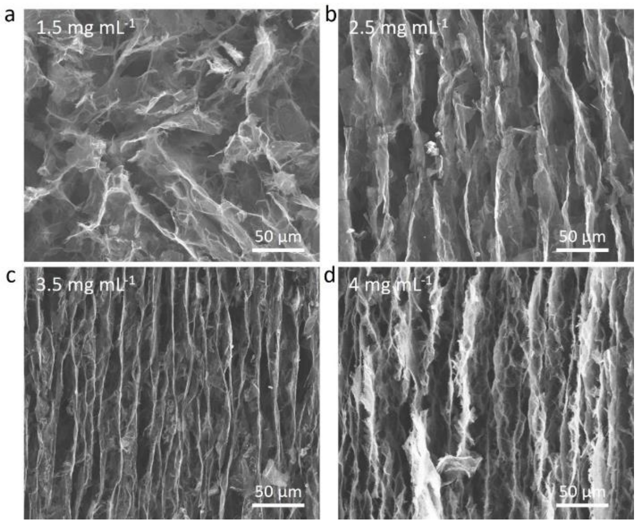

Supplementary Figure 2. SEM images of graphene assemblies with different initial GO concentrations. a, 1.5 mg mL^{-1}, **b,** 2.5 mg mL^{-1}, **c,** 3.5 mg mL^{-1}, **d,** 4 mg mL^{-1}.

Four different GO concentrations (1.5 mg mL^{-1}, 2.5 mg mL^{-1}, 3.5 mg mL^{-1}, 4 mg mL^{-1}) were selected, and the microstructures of the resulting hydrogels were characterized. The quantity of alkali added increases or decreases proportionally with the amount of GO. At a GO concentration of 1.5 mg mL^{-1}, the resultant graphene hydrogel exhibits an overall disordered state, with a gap size (pore size) of approximately 30-50 μm. At lower GO concentrations, the interactions between GO layers are weakened, and even with the addition of an appropriate amount of alkali, it remains challenging to form a long-range ordered liquid crystal phase, resulting in an unordered structure of the hydrogel.

With increasing GO concentration, uniform and ordered orientation structures can be achieved at concentrations of 2.5 mg mL^{-1}, 3.5 mg mL^{-1}, and 4 mg mL^{-1}. The disparity lies in the gap size: at lower concentrations, the spacing is larger (approximately 30-40 μm), whereas with increasing concentration, the interlayer spacing of the orientation structure gradually diminishes. At 4 mg mL^{-1}, the gap size is reduced to approximately 10 μm. It is noteworthy that the orientation structure of *cg-G* is remarkably uniform at a concentration of 3.5 mg mL^{-1}.

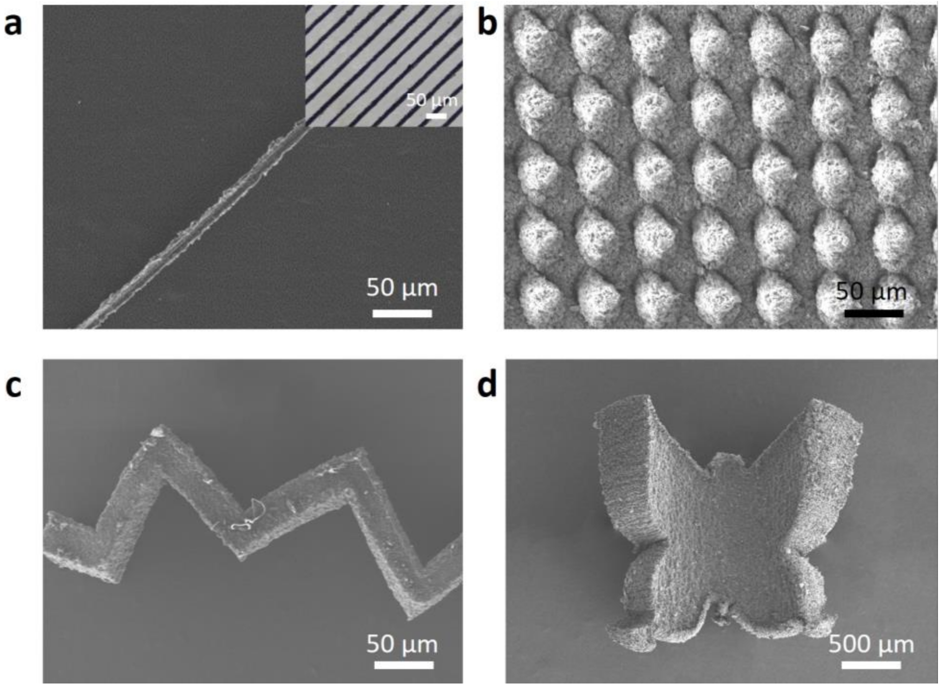

Supplementary Figure 3. Demonstrations of *cg-G* after evaporate-casting. SEM images of **a**, a stripe-shaped *cg-G* (illustration: photograph of stripe-shaped *cg-G*s array), **b**, point-shaped *cg-G*s array, **c**, zigzag-shaped *cg-G*, and **d**, butterfly-shaped *cg-G* after evaporate-casting.

The initial as-prepared *cg-G* is filled with water inside. In a natural setting, *cg-G* experiences dehydration, leading to spontaneous contraction. Through the utilization of laser sculpturing and following evaporate-casting process, it becomes possible to create various graphene microarchitectures such as stripe-shape, point-shape, zigzag-shape, and butterfly-shape, achieving an exceptional level of precision (~ 10 µm).

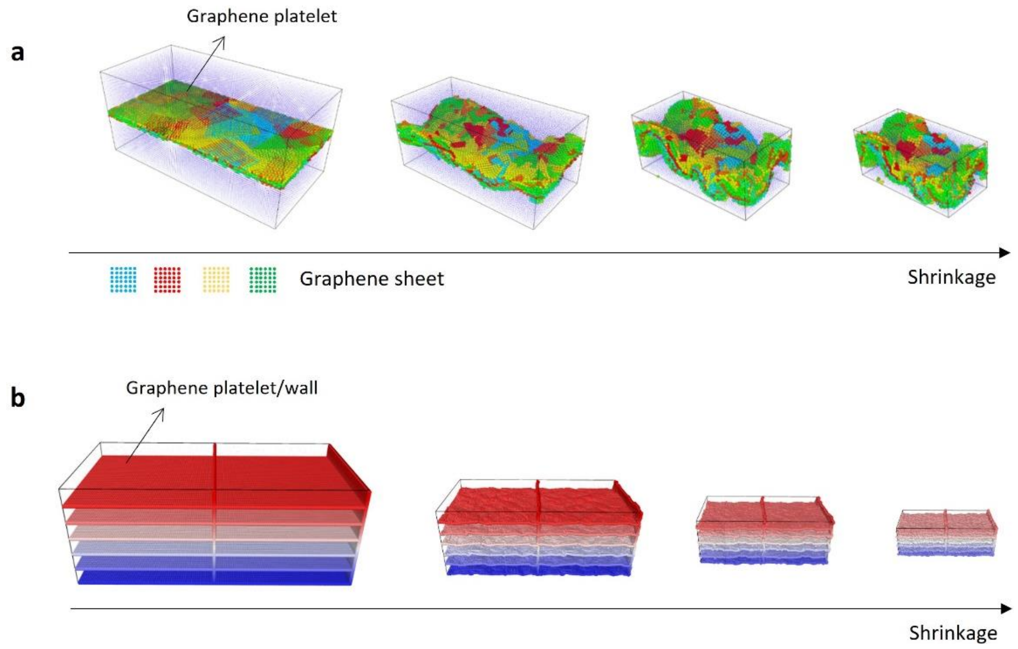

Supplementary Figure 4. CG-MD simulation of the evaporate-casting process of graphene walls in *cg-G*. a, Shrinkage behavior of a graphene platelet that contains multiple graphene sheets during evaporate-casting. The different colors in **a** represent different graphene sheets so that these sheets can be identified easily. As water molecules desorb gradually, the graphene platelet wrinkles. **b**, Shrinkage behaviors of simplified assembled graphene walls that consists of six oriented graphene platelets, which are denoted with different colors for the convenience of differentiation. It is found that by changing the bending modulus of the graphene wall, the wrinkling length changes accordingly.

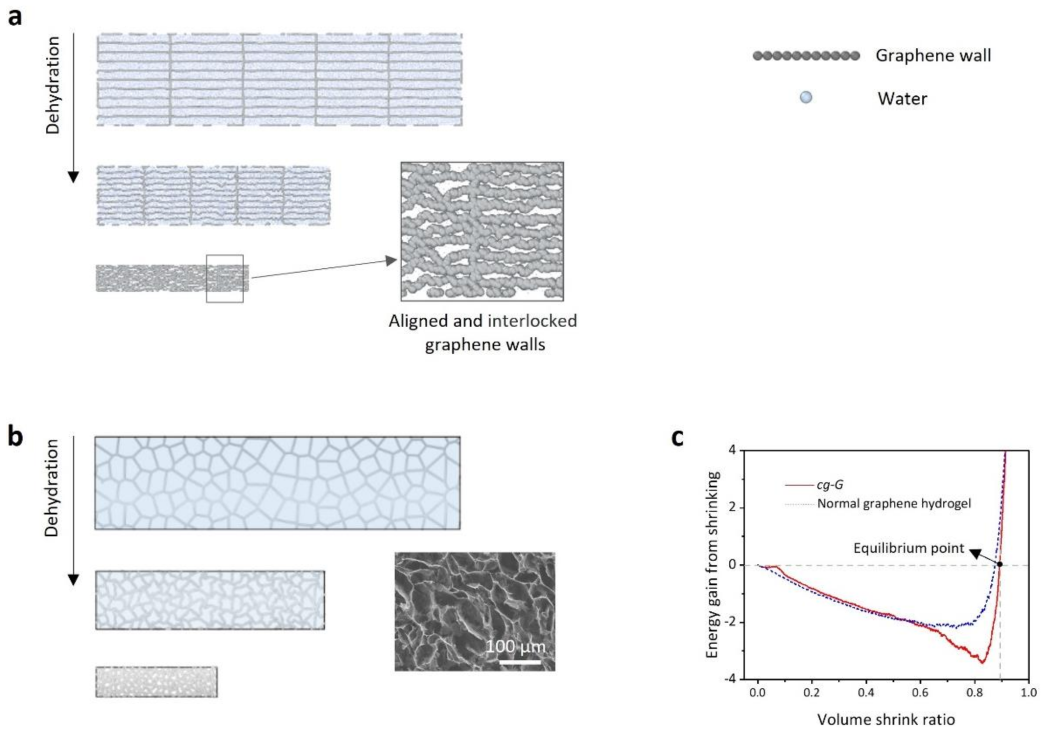

Supplementary Figure 5. CG-MD simulations of the evaporate-casting process of *cg-G* and normal graphene assembly. **a**, CG-MD simulation of the evaporate-casting process of *cg-G*. The simulation model is composed of graphene walls and water in them. The enlarged image shows the aligned and interlocked graphene walls under simulation. **b**, CG-MD simulation of the dehydration process of normal graphene hydrogel with uniform random microstructure. Illustration: SEM image of the normal graphene hydrogel before dehydration. **c**, Energy evolution curves of *cg-G* and normal graphene hydrogel during the dehydration process. Specifically, the energy gain from shrinking increases suddenly while the volume shrink ratio of the initial assembly exceeds 80% and reaches balance (energy gain from shrinkage is 0) with the volume shrink ratio of ~90%. The *cg-G* is much more energetically favorable than normal graphene hydrogel.

To unravel the underlying mechanism of the dehydration process, we conducted coarse-grained molecular dynamics (CG-MD) simulations, focusing on two phenomena at different scales. At first, we constructed a straight graphene platelet composed of multiple graphene sheets (Fig. S4a). As water molecules gradually reduce, the platelet tends to be wrinkled. Although our simulation successfully reproduces the

wrinkling phenomenon, its origin is hard to trace due to the geometrical complicacy of the model. Therefore, we employed a relatively simplified model (Fig. S4b) to study the origin of the wrinkling. It was found that by altering the bending modulus of the graphene wall, the length of the wrinkle changes accordingly. Like polymers, the graphene wall has a persistence length $L_p=B/(K_BT)$, which gives rise to the wrinkling phenomenon (where B is the bending stiffness, K_B is the Boltzmann constant, and T is the temperature).

The second phenomenon is the enhanced compactness and stability of graphene foam with ordered microstructures after dehydration compared to disordered ones. To investigate its origin, we established two configurations and simulated the evolution of their microstructures during water desorption (Fig. S5a, S5b). Under the influence of capillary forces, each parallel graphene wall contracts independently. The gap between them closes gradually and the convex edges interlock together, preserving the orientation microstructure of *cg-G*. This results in a dense yet aligned monolith. Energy analysis indicates that *cg-G* is more energetically favorable than normal graphene hydrogel (Fig. S5c). It is hypothesized that this phenomenon is attributable to the interference between adjacent walls during the contraction process. In normal graphene hydrogels, such interference prematurely halts the contraction process before the layers can attain their optimal state of contraction. In contrast, within ordered structures, individual walls contract independently first with minimal disruption. As more surfaces adhere to each other during dehydration, the energy decreases, contributing to the stability of the structure. As a result, *cg-G* can attain a more stable energy state and a more substantial degree of contraction.

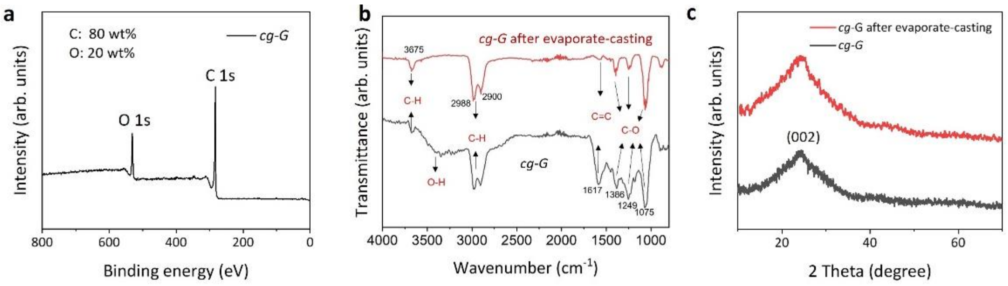

d

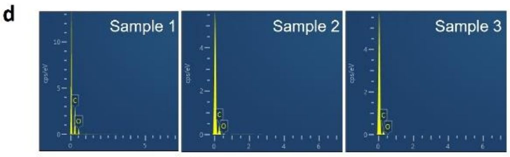

Samples	C (wt%)	O(wt%)
1	81.7	18.3
2	77.1	22.9
3	80.6	19.4
Average	79.8	20.2

e

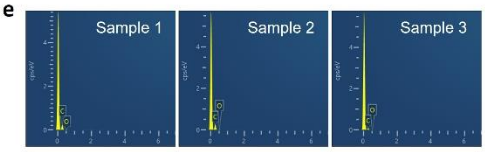

Samples	C (wt%)	O(wt%)
1	79	21
2	81.6	18.4
3	79.6	20.4
Average	80.1	19.9

Supplementary Figure 6. Materials characterizations of *cg-G*. a, XPS survey of *cg-G*. **b,** Fourier-transform infrared spectroscopy (FTIR) of *cg-G* before and after evaporate-casting. **c**, X-ray diffraction (XRD) patterns of *cg-G* before and after evaporate-casting. **d** and **e**, Energy dispersive spectroscopy (EDS) of *cg-G* before and after evaporate-casting.

As shown in Fig. S6a, *cg-G* displays a robust O 1s peak, with its oxygen atomic content measured at 20%. FTIR spectra of *cg-G* before and after evaporate-casting in Fig. S6b exhibit similar distinctive peaks. Specifically, the peaks at 3675 cm^{-1}, 2935 cm^{-1} and 2852 cm^{-1} are due to vibration of C-H bond stretching. The peak around 1617 cm^{-1} is attributed to sp^2-hybrided C=C stretches (in-plane vibrations). The peak at around 1075 cm^{-1}, 1249 cm^{-1}, and 1386 cm^{-1} correspond to C-O stretch. A broad peak at about 3400 cm^{-1}, which is related to the carboxyl O-H stretching mode of the presence of absorbed water molecules and alcohol groups, disappears in *cg-G* after evaporate-casting. It indicates that the pore size of *cg-G* significantly decreases after the evaporate-casting process, resulting in a reduction in the adsorbed water molecules and subsequently leading to the disappearance of the -OH peak.

The XRD patterns of *cg-G* before and after evaporate-casting exhibit nearly identical (002) peak in terms of both peak positions and half-peak widths. This observation strongly suggests that the interlayer distance of the graphene sheets within

individual graphene walls remain unaltered throughout the evaporate-casting process.

Fig. S6d and 6e show the EDS analysis results of *cg-G* before and after the evaporate-casting process. It is evident that the elemental composition and content of *cg-G* remained almost unchanged during the evaporate-casting process. After evaporate-casting, the surface of *cg-G* remains predominantly composed of C and O elements, with C constituting ~80 wt% and oxygen ~20 wt% of its composition. This is consistent with the FTIR data, affirming that the deformation process is a gentle, low-temperature transformation that would not alter its chemical composition.

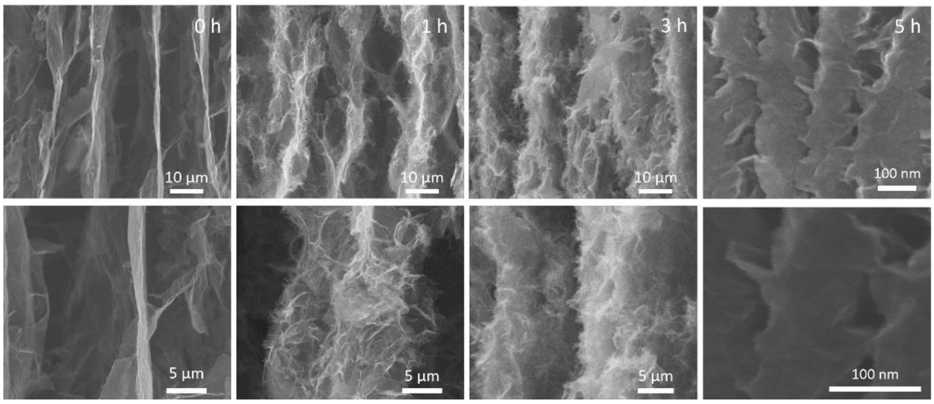

Supplementary Figure 7. SEM images of *cg-G* after evaporate-casting in different time (0 h, 1h, 3h, 5h). Initially, the graphene sheets within the *cg-G* hydrogel are aligned parallelly, exhibiting relatively thin sheets with approximately 20 μm spacing between them. As water within surface wrinkles evaporates, capillary forces prompt each graphene nanosheet to crumple. The reduction in distance between adjacent graphene sheets at this stage is not significant. With further evaporation, at the 3-hour mark, a substantial amount of water molecules between the graphene sheets are removed, leading to further wrinkling and densification of the graphene sheets, which draw closer together, resulting in a reduction in gap size. Finally, after 5 hours, when all water molecules have been completely eliminated, protrusions emerge on the dehydrated wall surfaces. A compact graphene superstructure with an oriented arrangement of graphene layers is achieved.

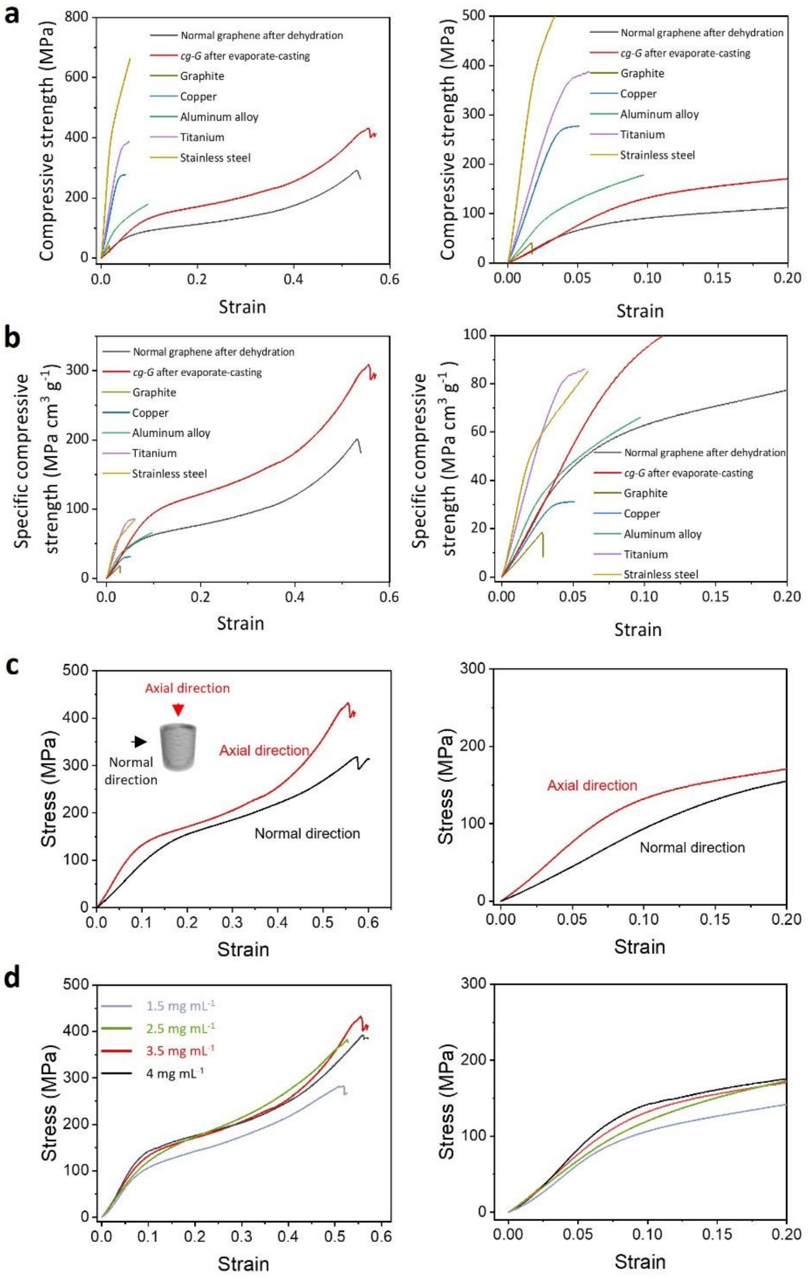

Supplementary Figure 8. The mechanical properties of the *cg-G* after evaporate-casting, other carbon materials, and various metals. a, Compressive stress-strain curves of the *cg-G* after evaporate-casting and other materials, and corresponding enlarged compressive stress-strain curves. **b**, Specific compressive stress-strain curves and corresponding enlarged specific compressive stress-strain curves. The *cg-G* after evaporate-casting demonstrates a higher specific compressive strength compared with various metals including stainless steel (SUS304) and titanium alloy (TC4). In addition, the *cg-G* after evaporate-casting shows plasticity like metals as well, showing a

promising application as structural parts. **c**, Compressive stress-strain curves of the *cg-G* after evaporate-casting under different compression directions, and corresponding enlarged compressive stress-strain curves. **d**, Compressive stress-strain curves of the *cg-G* with different initial GO concentrations after evaporate-casting, and corresponding enlarged compressive stress-strain curves.

As is evident from Fig. S8c, *cg-G* demonstrates notably high strength (~100 MPa) in two perpendicular stress directions. The difference lies in the fact that there is a greater Young's modulus along the direction of the sheets, while there is greater elastic and plastic deformation perpendicular to the direction of the sheets. This indicates favorable mechanical properties in both directions, allowing the loading surface to be selected based on specific needs in practical applications

As illustrated in Fig. S8d, we conducted a thorough examination of the mechanical properties of graphene hydrogels after evaporate-casting with various initial GO concentrations. Notably, the mechanical superiority of *cg-G* superstructures over disordered graphene assemblies, as discussed in Fig. 2g of the revised main text, is evident. This discrepancy arises from the influence of capillary forces induced by dehydration during the evaporate-casting process, which drive the deformation of the graphene assembly. At the nanoscale, graphene nanosheets form protruding structures, creating mechanical interlocks. At the assembly level, the layered structure exhibits enhanced orientation and stacking, resulting in extensive contact areas and intensified interlayer interaction. In contrast, disordered graphene assemblies only demonstrate mechanical reinforcement at the nanoscale, leading to inferior mechanical properties compared to *cg-G* superstructures. Additionally, all samples of the other three types of *cg-G* superstructures exhibited exceptional mechanical compression performance.

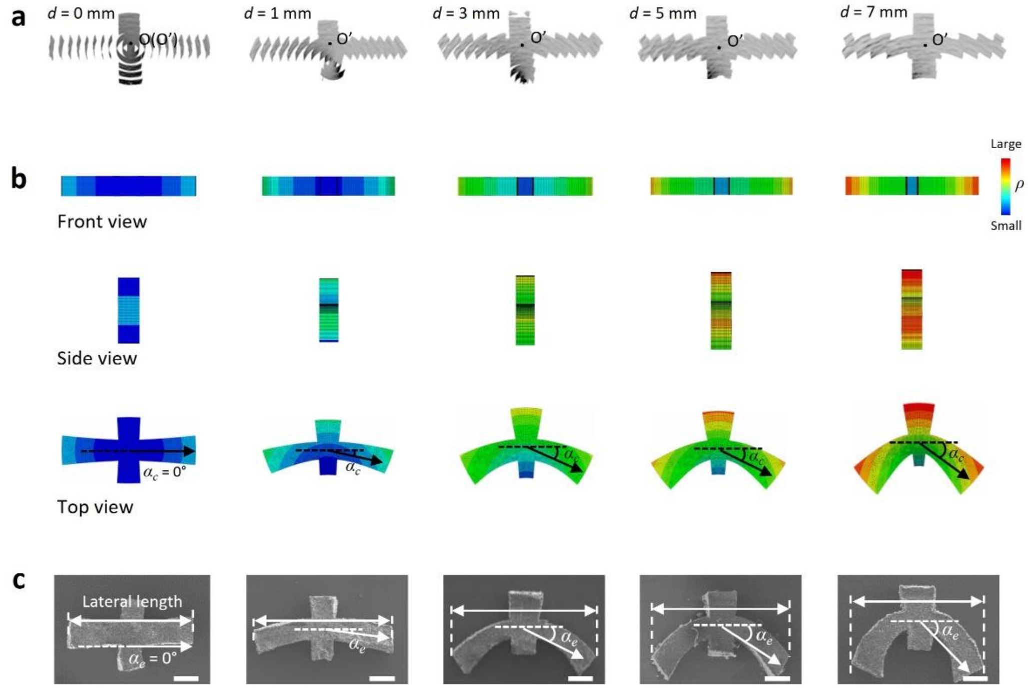

Supplementary Figure 9. In-plane processing: demonstrations of cross-shaped *cg-Gs* with different microstructures before and after deformation. a, Schematic diagrams of five cross-shaped *cg-G*s with different axis-center distances (d) perpendicular to centric axis of curvature. **b**, Gradient distribution of the curvatures and theoretical predictions of the deformation of the five *cg-G*s (d = 0, 1, 3, 5, 7 mm) based on FEA. **c**, SEM images of the five *cg-G*s after evaporate-casting. The colors in FEA from blue to red indicate an increase of the radius of curvature (ρ). The obtained assemblies demonstrated obvious deformations compared with their initial profiles. The degree of deformation can be represented by the included angle (α_e). Scale bar: 500 μm. With the increase of d, the angle α_e increases as well. The experimental results are very close to that of calculated values in FEA.

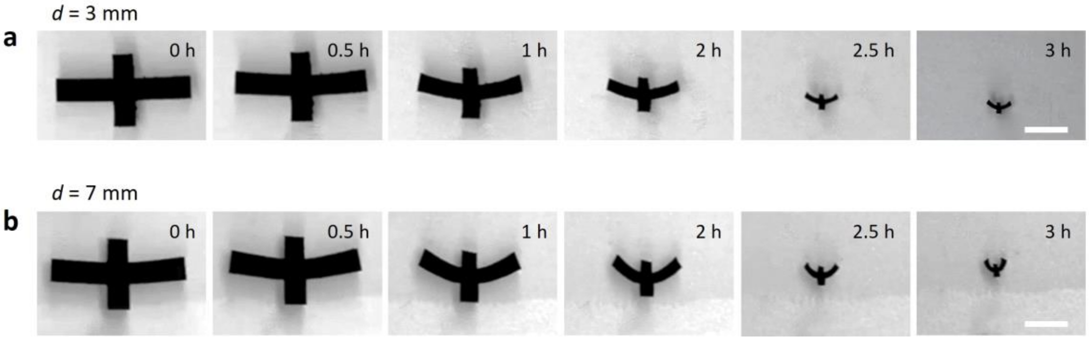

Supplementary Figure 10. Photographs of the deformation processes of cross-shaped *cg-G*s with different axis-center distances. a, *d* = 3 mm. **b**, *d* = 7 mm. Scale bar: 5 mm. A larger axis-center distance results in a more noticeable deformation.

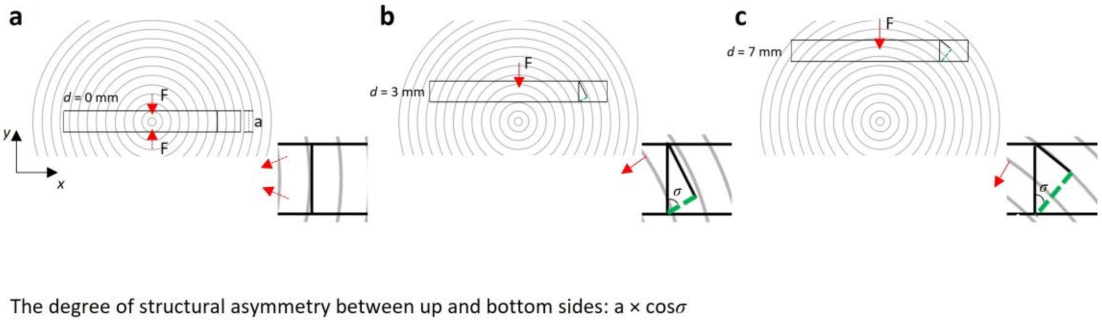

Supplementary Figure 11. Schematics of the structural asymmetry of stripe-shaped *cg-G*s with different *d* values. a, *d* = 0 mm. **b**, *d* = 3 mm. **c**, *d* = 7 mm. The width of the sample is *a*, the angle between the normal of the graphene wall (dotted green line) and *y*-axis is *σ*. The degree of structural asymmetry between the up and bottom sides can be expressed as $a \times \cos \sigma$. With increasing *d* value, the angle also enlarges, resulting in a heightened degree of structural asymmetry. That is, the prominence of the differential distribution of the shrinkage coefficient amplifies as *d* increases.

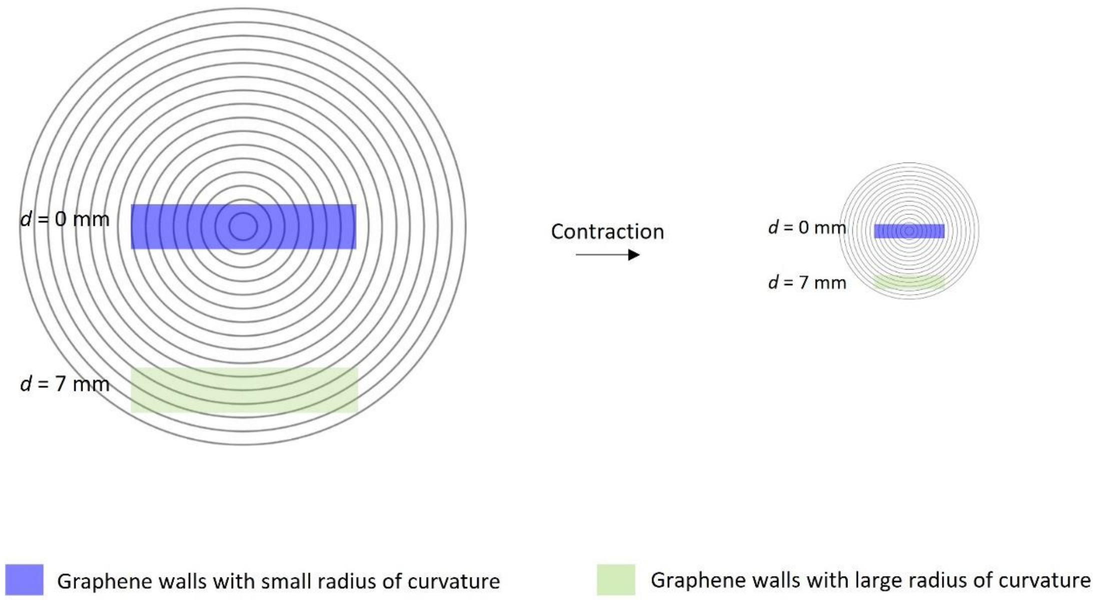

Supplementary Figure 12. Schematic of two stripe-shaped *cg-G*s with different microstructures in an ideal coaxial circular arc model. The *cg-G* of *d* = 0 mm (blue) mostly consists of arcs with small radii and gaps between them, while the *cg-G* of *d* = 7 mm (green) mostly consists of arcs with large radii. In an ideal coaxial circular arc model, the assembly tends to contract uniformly. Various curvature structures within assemblies demonstrate consistent lateral dimensions following contraction deformation.

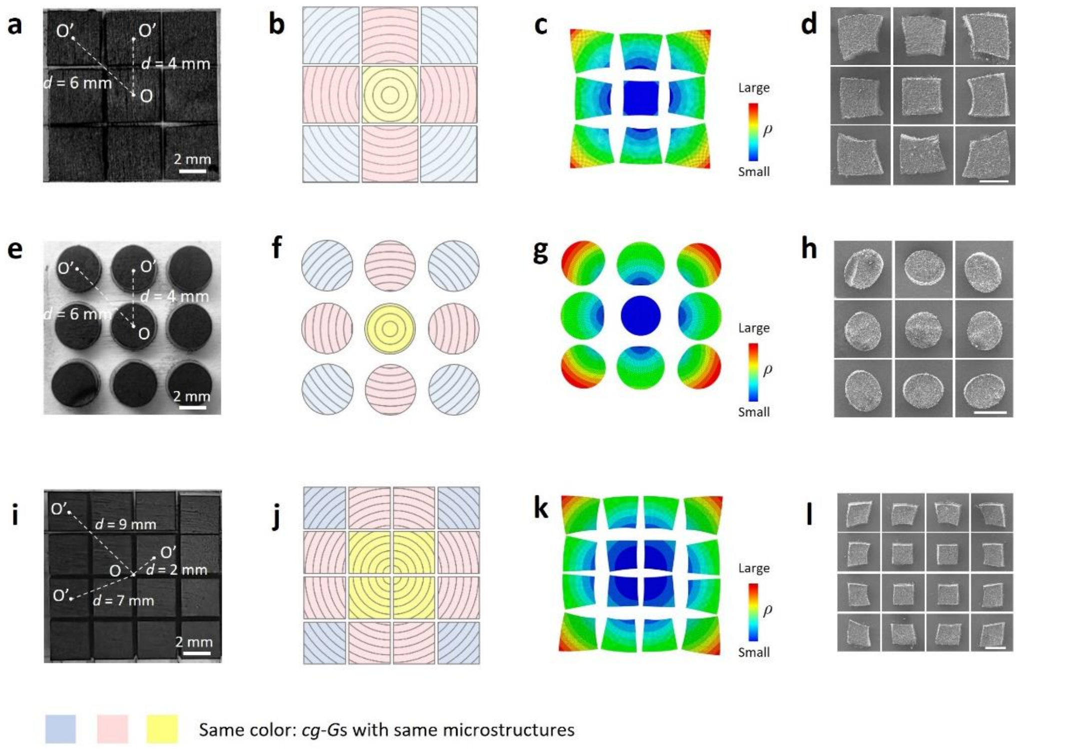

Supplementary Figure 13. In-plane evaporate-casting process of *cg-G*s with different configurations and *d* values. a, Photographs of square-shaped *cg-G*s distributed in a nine-grid pattern. **b**, Schematic of the microstructures within those square-shaped *cg-G*s. **c**, Gradient distribution of the curvatures and theoretical predictions of the deformation based on FEA and **d**, SEM images of these square-shaped *cg-G*s after evaporate-casting. **e**, Photographs of circular *cg-G*s distributed in a nine-grid pattern, and corresponding **f**, schematic of the microstructures. **g**, FEA results and **h**, SEM images of these circular *cg-G*s after evaporate-casting. **i**, Photographs of square-shaped *cg-G*s distributed in a 4 × 4 grid pattern, and corresponding **j**, schematic of the microstructures. **k**, FEA results and **l**, SEM images of these square-shaped *cg-G*s after evaporate-casting. Scale bars in **d**, **h**, **l**: 500 μm. The colors in FEA from blue to red indicate an increase of the radius of curvature (ρ).

As illustrated by Fig. S13, all *cg-G*s located away from the center display in-plane deformations towards the curvature center direction following the evaporate-casting process, and the degree of deformation increases with an increasing *d* value. In contrast, the microstructure of the *cg-G* at the central position is centrally symmetric and retains its initial shape perfectly after undergoing the water casting process.

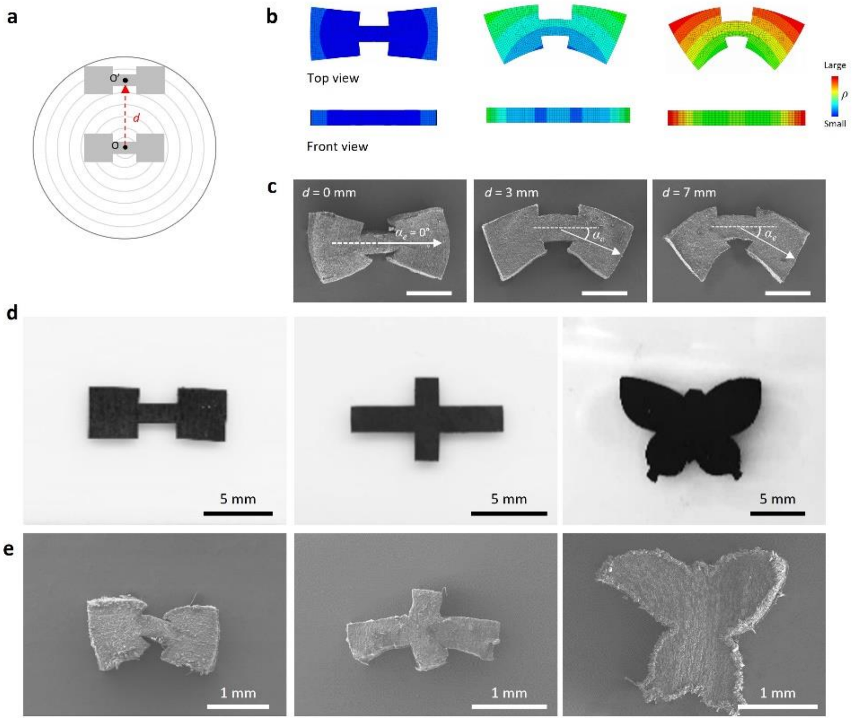

Supplementary Figure 14. In-plane evaporate-casting process. **a**, Schematic diagram of dumbbell-shaped *cg-G* with different *d* values derived from concentric circle structure. **b**, Gradient distribution of the curvatures and theoretical predictions of the deformation based on FEA and **c**, SEM images of the three dumbbell-shaped *cg-G*s (d = 0, 3, 7 mm) after evaporate-casting. Scale bar: 1 mm. The degree of deformation can be represented by the included angle (α_e). The colors in FEA from blue to red indicate an increase of the radius of curvature (ρ). The dumbbell-shaped *cg-G*s exhibits a deformation trend similar to the cross-shaped *cg-G*s. Digital and SEM images of normal graphene hydrogels with different geometric structures **d**, before and **e**, after contraction.

For the geometric structure of normal graphene hydrogels, the contraction process entails a reduction in volume with irregular altering in the shape. This is attributed to the irregular nature of the internal structure of normal graphene hydrogels. During the shrinkage process, the local asymmetric microstructure causes internal stress, which leads to uncontrollable deformation.

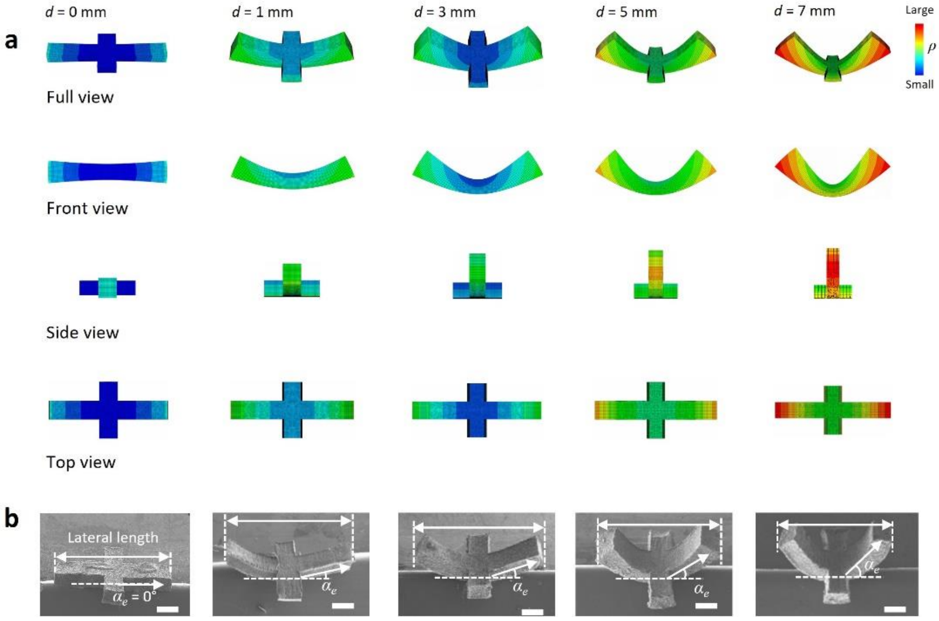

Supplementary Figure 15. Out-plane evaporate-casting processing: demonstrations of cross-shaped *cg-G*s with different *d* values after deformation. a, Gradient distribution of the curvatures and theoretical predictions of the deformation of the five *cg-G*s (*d* = 0, 1, 3, 5, 7 mm) based on FEA. **b**, SEM images of the five *cg-G*s after evaporate-casting. The colors in FEA from blue to red indicate an increase of the radius of curvature (ρ). The obtained assemblies demonstrate obvious deformations compared with their initial profiles. The degree of deformation can be represented by the included angle (α_e). Scale bar: 500 μm.

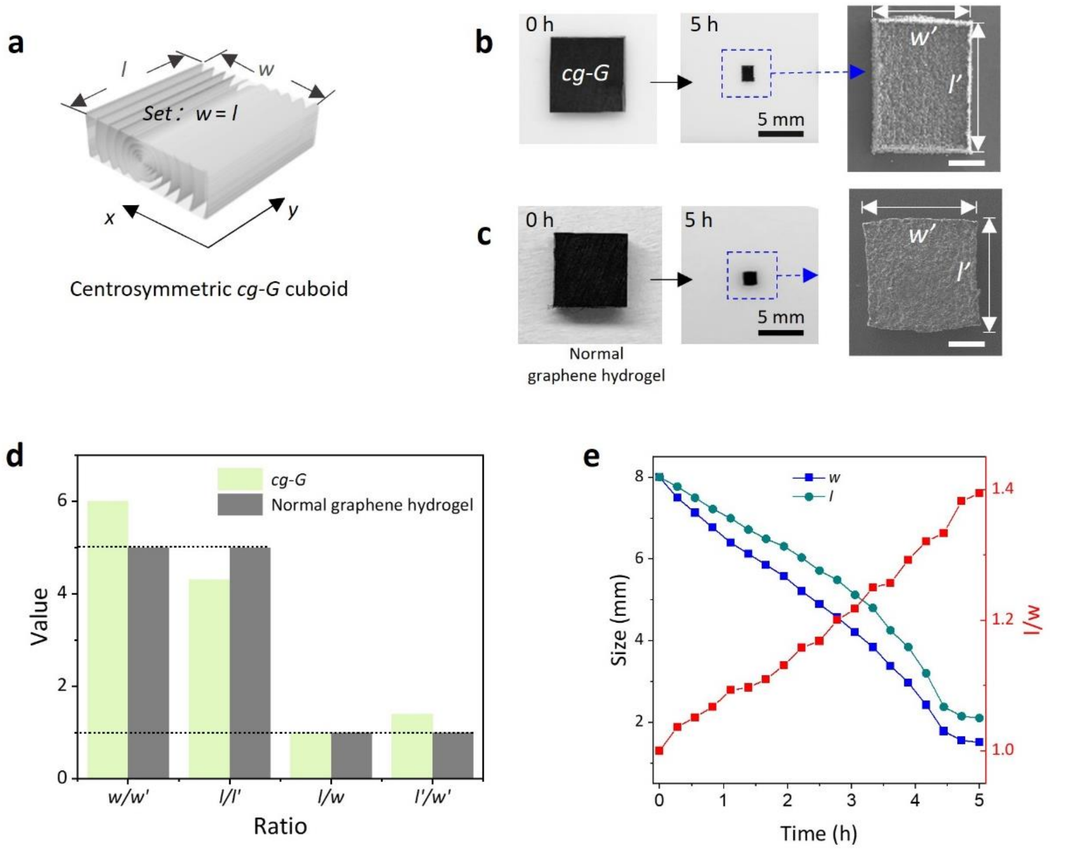

Supplementary Figure 16. Mechanism of evaporating-casting of *cg-G*. a, Schematic of cuboid-shaped *cg-G* with centrosymmetric curvature gradient microstructure. Digital photos of **b**, the cuboid-shaped *cg-G* and **c**, normal graphene hydrogel with random microstructure after dehydration of 0 h and 5 h, and corresponding SEM images for 5 h of dehydration. Initial size: 8 mm × 8 mm × 2 mm. The lengths of the sides along and perpendicular to the direction of orientation microstructure before and after dehydration are named *l*, *w*, and *l'*, *w'*, respectively. **d**, The ratio values of *w*/*w'*, *l*/*l'*, *l*/*w* and *l'*/*w'* of *cg-G* and normal graphene hydrogel. **e**, The size change of *l* and *w* and the ratio change of *l*/*w* for *cg-G* during the water evaporation process.

Fig. S16a is the schematic of a cuboid-shaped *cg-G* with centrosymmetric curvature gradient structure. In the *y*-axis direction, *cg-G* consists solely of continuous graphene walls, while in the *x*-axis direction, *cg-G* comprises graphene walls separated by gaps. As shown in Fig. S16b, the square plane of the cuboid-shaped *cg-G* transformed into a rectangle after dehydration, indicating the different deformation ratios and rates along the two axes. Specifically, the ratio of *w*/*w'* was 6, much higher

than the ratio of *l*/*l'* at 4.3 (Fig. S16d). Additionally, the aspect ratio of *l'*/*w'* reached up to 1.4. This result is expected to vary with the initial gap size, while larger gaps would result in a higher *l'*/*w'* ratio. On the contrary, the normal graphene hydrogel after dehydration retained its initial profile, with an *l'*/*w'* aspect ratio of 1.

Furthermore, during evaporating, deformation rates of all sides initially increased and then decreased (Fig. S16e). This can be attributed to the decreasing volume of the hydrogel slice, which leads to a continuous increase in the specific surface area and subsequently accelerates the evaporation rate. This process continues until all the water is depleted and the deformation process concludes. Additionally, the larger slope of the curve for side *w* compared to side *l* means a greater deformation rate for the gap side, and the ratio of *l*/*w* increases proportionally over time.

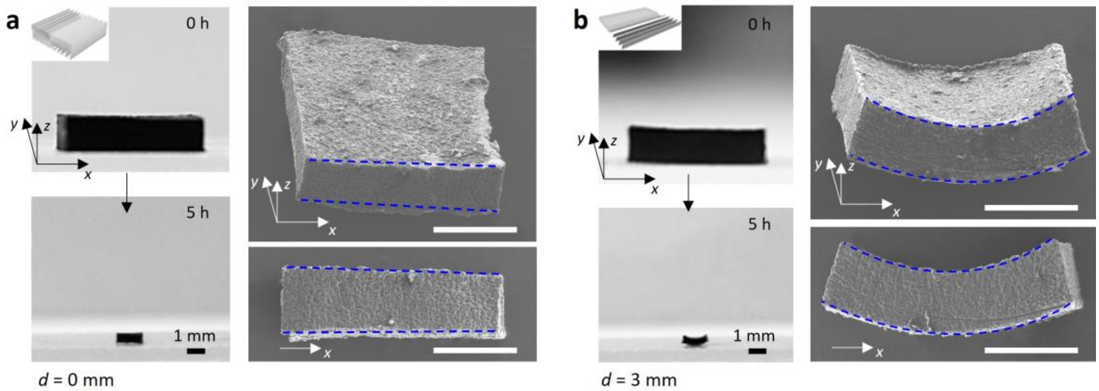

Supplementary Figure 17. Verification of out-plane processing. **a**, Digital images of the dehydration process (from 0 h to 5 h) of *cg-G* cuboid (side view) (8 mm × 8 mm × 2 mm) at center axis with the microstructure shown in illustration, and corresponding SEM images. No out-plane deformation can be observed. Scale bar: 500 µm. **b**, Digital images of the dehydration process (from 0 h to 5 h) of *cg-G* cuboid (side view) (8 mm × 8 mm × 2 mm) off center axis with the microstructure shown in illustration and corresponding SEM images. Out-of-plane bending deformation occurs, which looks like a tile. This deformation phenomenon aligns with the discussion presented in Fig. 3. Scale bar: 500 µm.

It is obvious that none apparent out-plane deformation of the center vertical slice could be detected from the photographs and SEM characterization (Fig. S17a). The reason is that the configurational force occurring in the slice is cancelled out due to the symmetric microstructures on *z*-axis. Scilicet, the *cg-G*s off center demonstrate different phenomenon on *z*-axis. As shown in Fig. S17b, the *cg-G* can be expressed as the combination of graphene walls with large radius of curvatures near the upper side and small radius of curvatures near the bottom side. The asymmetric microstructure on *z*-axis results in the different deformation degree of up and bottom sides, and corresponding asymmetric configurational force. As a result, two sides of *y*-axis were dragged up by the configurational force, and leading to a finial graphene architecture bended look like a tile.

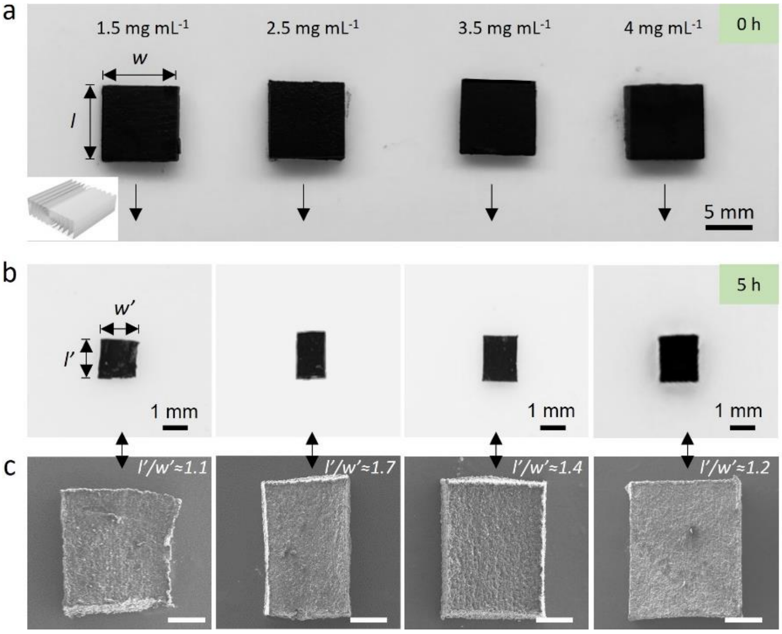

Supplementary Figure 18. Characterizations of the *cg-G* microstructure's impact on the evaporate-casting process. a, b) Photographs of cuboid-shaped graphene hydrogels (with different initial GO concentrations) with centrosymmetric curvature gradient microstructure after dehydration of 0 h and 5 h, and (**c**) the corresponding SEM images of dehydration for 5 h. Scale bar: 500 μm. Initial size: 8 mm × 8 mm × 2 mm. The ratios of the two sides along (*l'*) and perpendicular to the wall plane (*w'*) after dehydration are different.

To investigate the impact of the initial gap size in the *cg-G* on deformation behavior, we opted cuboid-shaped graphene hydrogels with centrosymmetric microstructures at different initial GO concentrations. The initial configuration, dimensions, and microstructure of the cuboid-shaped hydrogels are illustrated in Fig. S18a.

For the initial hydrogels, the ratio of *l* to *w* is 1. Following 5 hours of spontaneous water evaporation, all four cuboid-shaped hydrogels underwent contraction and deformation. As shown in Fig. S18b and S18c, it can be observed that the deformation behaviors of the four hydrogels vary significantly. Among them, the hydrogel with an initial GO concentration of 1.5 mg mL^{-1} underwent irregular contraction. As the GO concentration increased, the regularity of the *cg-G* significantly improved after

evaporate-casting, with the degree of contraction of the *w* side being noticeably greater than that of the *l* side, resulting in *l'/w'* > 1. The higher the initial GO concentration, the smaller the *l'/w'* ratio. The values of *l'/w'* corresponding to the three GO concentrations are 1.7, 1.4, and 1.2, respectively. As discussed in the main text, this indicates an anisotropic contraction behavior that could significantly enhance the effectiveness of curvature gradient-induced deformation. Furthermore, the enhancement of deformation behavior diminishes with increasing initial GO concentration.

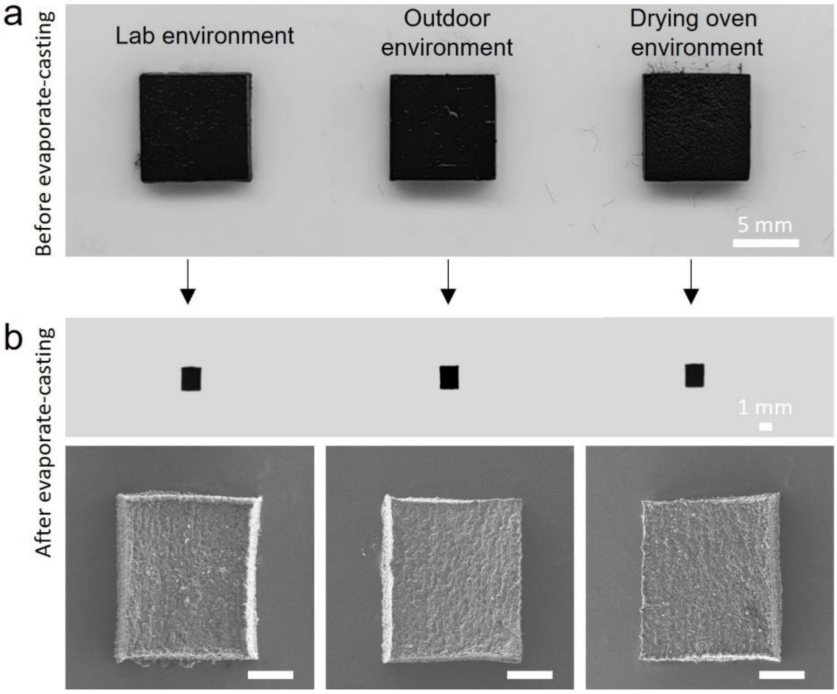

Supplementary Figure 19. Characterizations of *cg-G* **a**) before and **b**) after evaporate-casting with different drying method. Scale bar: 500 μm.

To investigate the effects of different drying methods on *cg-G* evaporate-casting behavior, we selected three *cg-G* hydrogel samples with identical internal orientation microstructures. These samples were subjected to drying outdoors, convection oven, and drying in the laboratory environment, respectively. The outdoor drying occurred with a temperature of approximately 23 °C and a northwest wind of 3 levels. The convection oven was set at 35 °C with forced air circulation.

After the evaporate-casting process, all three *cg-G* superstructures exhibited almost identical configurations and dimensions. This indicates that factors such as air flow velocity and temperature, which can alter the evaporation rate of water, do not affect the outcome of the evaporate-casting deformation behavior. However, it was observed that the time required for evaporation deformation varied significantly across different environments, which demonstrates that different drying methods only affect the timing of the deformation process without impacting the final structure.

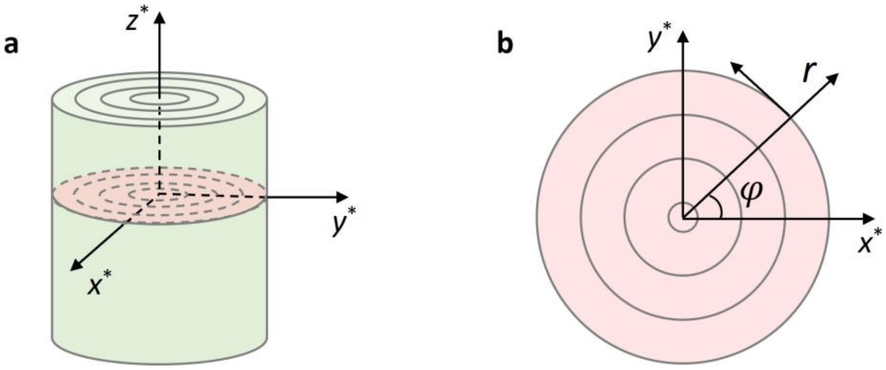

Supplementary Figure 20. The coordinate system in theoretical analysis. a, Schematic of graphene walls composed of multiple sets of "cylinders" with different curvatures. The coordinate axis is denoted by x^*-y^*-z^*. **b**, Schematic of x^*-y^* plane of **a**. r is the distance to the cylinder's axis. φ represents the angle to x^*-axis.

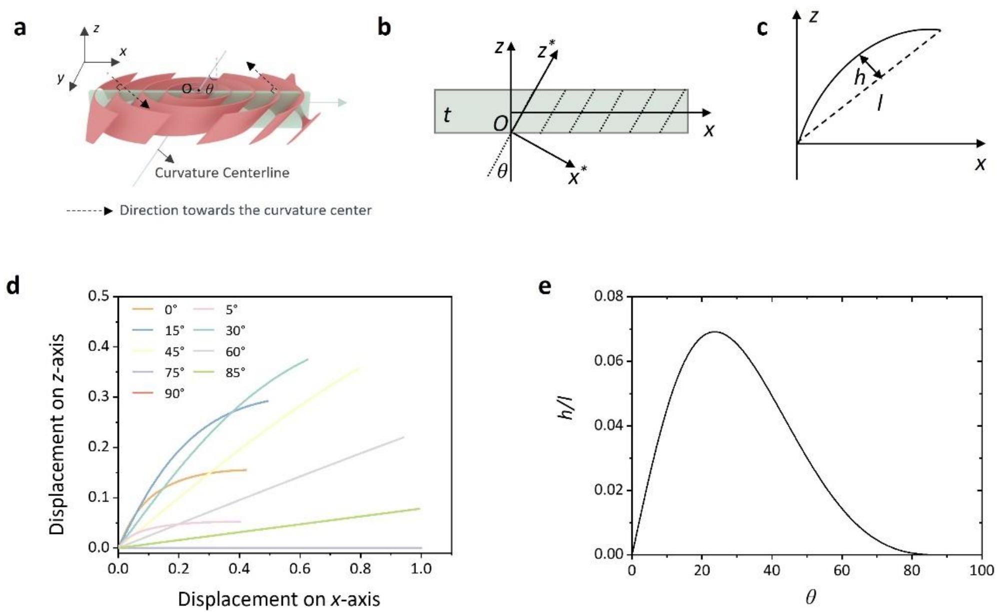

Supplementary Figure 21. The coordinate system in theoretical analysis. Schematic diagrams of **a**, complex *cg-G* and corresponding slice of **b**, *x-z* face, which represents strip used in the model analysis (green slice in **a**). *z-y-z* represents the new coordinate axis of the sample. The thickness of the *x-z* slice is defined as *t*. The angle between graphene wall and the normal to the sample plane is defined as θ. The schematic diagrams of these representative strips are shown in **b**. Dashed lines represent the graphene walls. x^*-z^* represent the coordinate axis of initial cylinder. **c**, Define the degree of deformation through the height and bottom of the curve. *l* represents the length of the bottom of the graphene slice after deformation. *h* represents the largest height of the bended graphene slice. **d**, The shapes of the deformed samples. The shape is regulated by the angle θ. **e**, The dependence between the degree of deformation and the angle θ. The degree of deformation is expressed by *h/l*. The larger the *h*, and the smaller the *l*, the greater the degree of deformation of the graphene wall.

To further confirm the reliability of the formula, we investigated the deformation of a more intricate curvature gradient structure with two different directions toward the curvature center. The deformation of this intricate structure occurs on three faces, namely: *x-z*, *y-z*, and *x-y* faces. Firstly, an analysis is conducted on the slender strip at "*y*=0" within the sample (*x-z* face, Fig. S21a, Fig. S21b). General case of a cross section of *cg-G* passing through the curvature center is characterized by the thickness of the slice *t*, the position on *x*-axis *x*, and the angle between graphene wall and the normal to

the sample plane θ. During the dehydration process, the sample undergoes strains pointing towards the center of curvature, and the magnitude of these strains is related to the curvature radius of the graphene wall at that location. Due to the unequal curvature of graphene walls on the upper and lower surfaces of the sample, the sample experiences bending deformation (Fig. S21c). The displacement of various points within the sample is as follows:

$$u_x = (x\varepsilon_0 \cos\theta + \frac{1}{3} bx^3 (\cos\theta)^3) \cos\theta \quad (1)$$

$$u_z = -(x\varepsilon_0 \cos\theta + \frac{1}{3} bx^3 (\cos\theta)^3) \sin\theta \quad (2)$$

Where u_z is the displacement on z direction, u_x is the displacement on x direction. ε_0 (< 0) represents the uniform shrinkage strain (the curvature radius equals zero). b (>0) is the curvature deformation coefficient, indicating the sensitivity of deformation to changes in curvature radius. The maximum deformation of the sample occurs at ~23° (Fig. S21e).

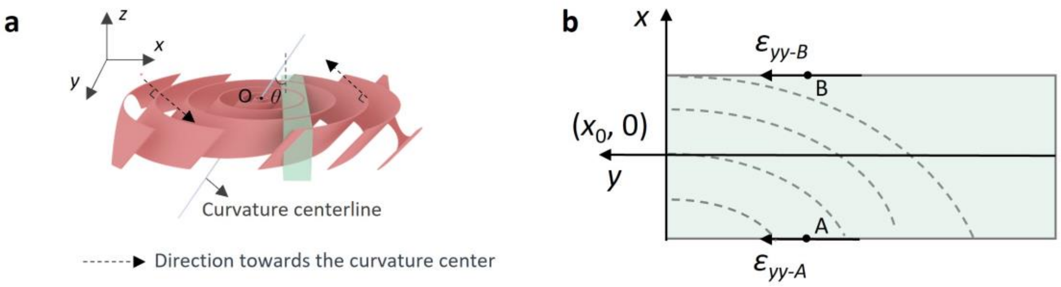

Supplementary Figure 22. The coordinate system in theoretical analysis. Schematic diagrams of **a**, complex *cg-G* and corresponding slice of *x*-*y* face (green slice), which represents strip used in the model analysis. **b**, The schematic diagrams of these representative strips are shown. Dashed lines represent the graphene sheets. The position of the center slice of *x*-*z* face on *x*-axis is defined as x_0. **a** and **b** are reference points on the surface. ε_{yy-A} and ε_{yy-B} respectively denote the projections of strains at points A and B on the upper and lower surfaces of the sample along the *y*-axis.

We employ the same method in Fig. S21 to study the deformation of the configuration within the *y*-*z* and *x*-*y* plane. An expression about displacement on *x*-*y* (u_x) plane can be given as follow:

$$u_x = -\frac{1}{3} b x_0 y^2 \cos^2\theta \tag{3}$$

Where x_0 is transverse coordinate of the sample, which is equivalent to the distance *d* in experiment. It can be observed that as the slice moves away from the center of the cylinder, the sample exhibits greater bending deformation in the *x*-*y* plane. When the graphene wall is perpendicular to the sample, i.e., $\theta = 0°$, the bending deformation in the *x*-*y* plane is maximized. As θ increases, the bending deformation gradually decreases until it reaches zero.

The expression about displacement on *y*-*z* plane (u_z) can be given as follow:

$$u_z = \frac{1}{3} b x_0 y^2 \cos\theta \sin\theta \tag{4}$$

From the above equation, it is evident that the bending deformation in the *y*-*z* plane reaches its maximum value at $\theta = 45°$.

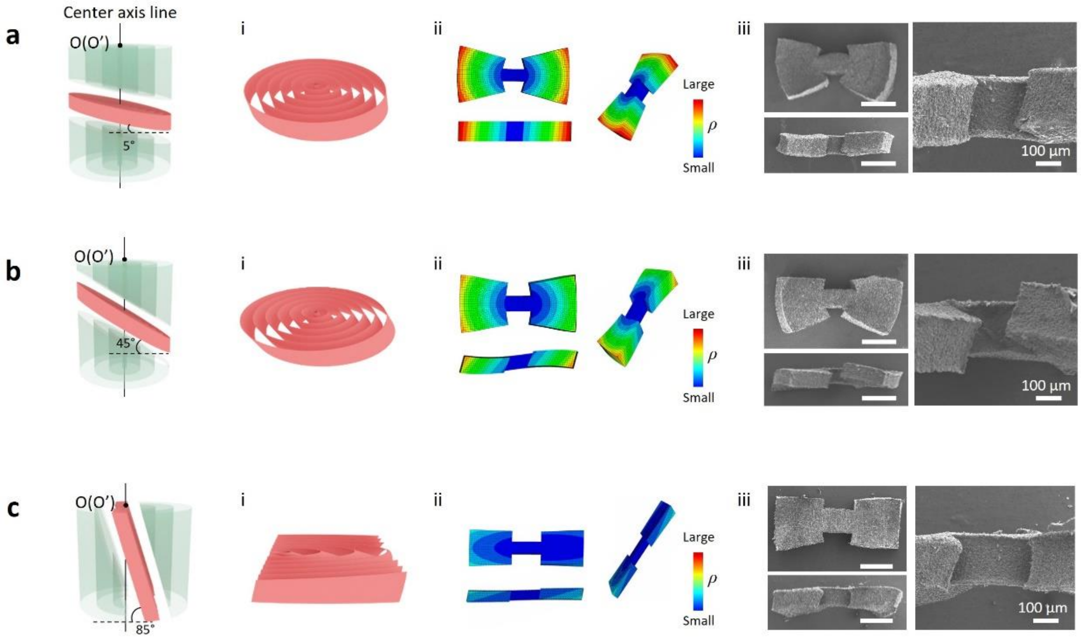

Supplementary Figure 23. Verification of the self-morphing mechanistic models of *cg-G*. a-c, Simplified schematic of complex *cg-G* with θ of 5°, 45°and 85°, and (i) corresponding schematics of detailed microstructures. (ii) Gradient distribution of the curvatures and theoretical predictions of the deformation based on FEA and (iii) SEM images of the three dumbbell-shaped *cg-G*s after evaporate-casting. The colors in FEA from blue to red indicate an increase of the radius of curvature (ρ). Scale bar: 500 µm.

Take θ of 5°, 45°, and 85° as examples, the FEA results and SEM images indicate that the architecture twists around the long center axis after deformation, resulting in a stereo and centrosymmetric final geometry, and bilateral cuboids transformed to be approximate trapezoidal cube due to the in-plane deformation. The deformation curve of the long center axis for samples with different θ closely mirrors the shape depicted in Fig. S21d. In addition, the maximum value of in-plane deformation is obtained at 5°, while the *cg-G* with tilt angle of 45° demonstrates the largest out-plane (*y-z*) deformation. These results are in perfect agreement with the speculated deformation tendency.

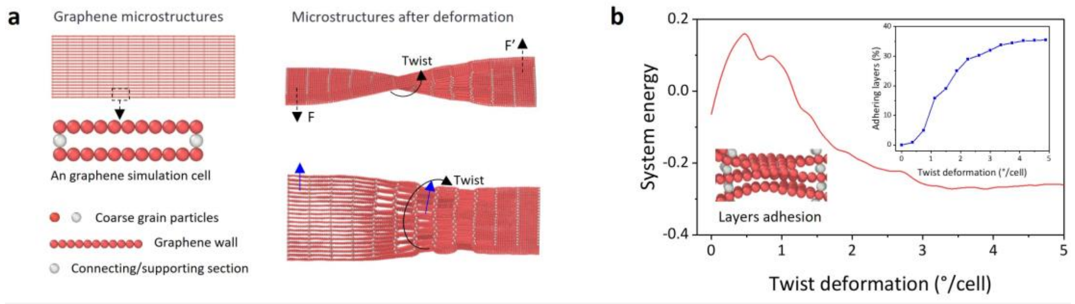

Supplementary Figure 24. CG-MD simulation of the *cg-G* under twist deformation. **a**, CG-MD simulation of the microstructures of *cg-G* before and after being twisted under external force. This simulation model comprises numerous graphene simulation cells. Under external forces, the assembly undergoes twist deformation, resulting in a shift in the direction of the curvature center of the walls (blue arrow). **b**, Energy evolution and the percentage of graphene walls adhering to each other (illustration in upper right) of the simulation model during twisting process. Illustration in left bottom: adhesion of graphene walls after being twist.

CG-MD calculation model was established to simulate the changes of curvature gradient of *cg-G* under external force (Fig. S24a). Under the twist deformation, the direction of the curvature center of simulated graphene walls undergoes a modification. Meanwhile, the energy of the system initially increases and then decreases as raise of twist angles, indicating that this specific structure can absorb external energy during deformation, thereby enabling the system to reach a stable state (Fig. S24b). This phenomenon occurs because graphene walls draw nearer to one another during the deformation process. The strong π-π interactions lead to graphene walls adhering to each other (Fig. S24b, illustration in left bottom), subsequently lowering the overall energy of the system.

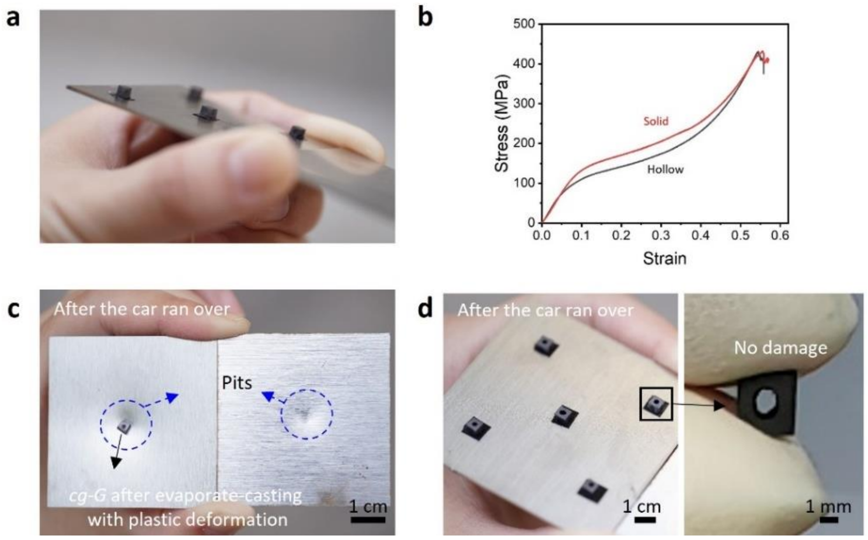

Supplementary Figure 25. Photographs of evaporate-casted *cg-G*s after being run over by car. a, Photograph of evaporate-casted solid *cg-G*s after being run over by car. After being run over by a car, no damage can be found on the five micro-fixators of *cg-G* after evaporate-casting. **b,** Compressive stress-strain curves of the solid and hollow *cg-Gs* after evaporate-casting. The stress-strain curve of the hollow one exhibits a similar shape and magnitude to that of the solid one. **c**, After being run over by a car, one micro-fixator of *cg-G* after evaporate-casting undergoes plastic deformation, and a pit is evident on both the upper and lower stainless steel plates. **d**, After being run over by a car, no damage can be found on the five micro-fixators of *cg-G* after evaporate-casting.

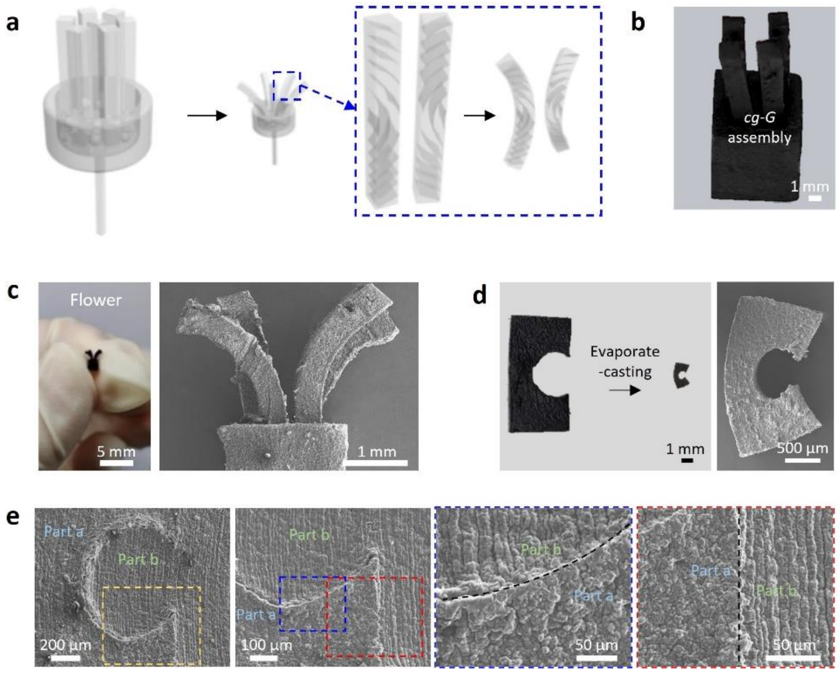

Supplementary Figure 26. Demonstrations of integrated *cg-G*s. a, Schematic of combined *cg-G*s mimicking the flowering process. The "petals" are composed of graphene walls with asymmetric curvature gradient structure. During evaporate-casting process, these petals can bend outward, which look like a flower in bloom. **b**, Photograph and **c**, SEM image of the combined *cg-G*s flower after evaporate-casting. **d**, Digital images of mechanically interlocked structures (part a) before and after deformation, and corresponding SEM image of mechanically interlocked structures (part a) after deformation. **e**, SEM images of the bonding interface of the mortise and tenon structure with different magnifications.

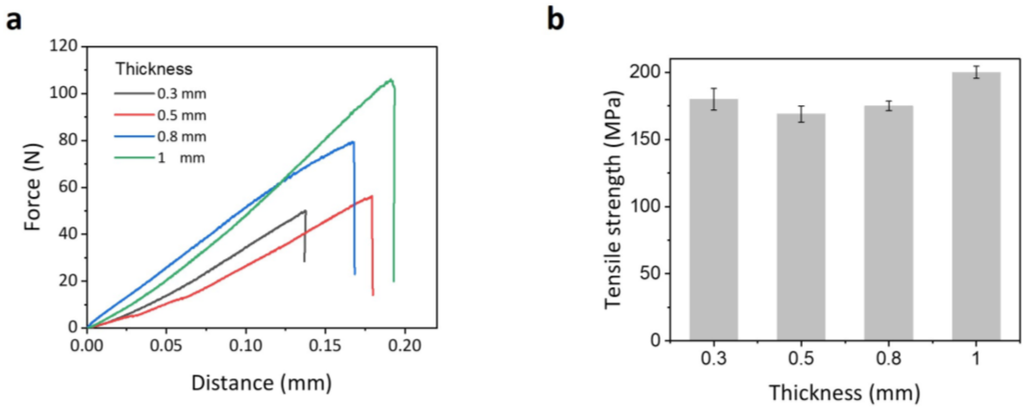

Supplementary Figure 27. Connection strength of *cg-G*s with mechanical interlocking structure after evaporate-casting. a, Tensile curves of connected *cg-G*s with different thicknesses. The fracture occurs in a position where both parts are locked. The distance just means the stainless steel wire is stretched, and has little to do with the stretching of graphene interlocking structures. **b**, Tensile strength of mechanical interlocking structures with different thicknesses (n = 3, error bar: standard deviation).

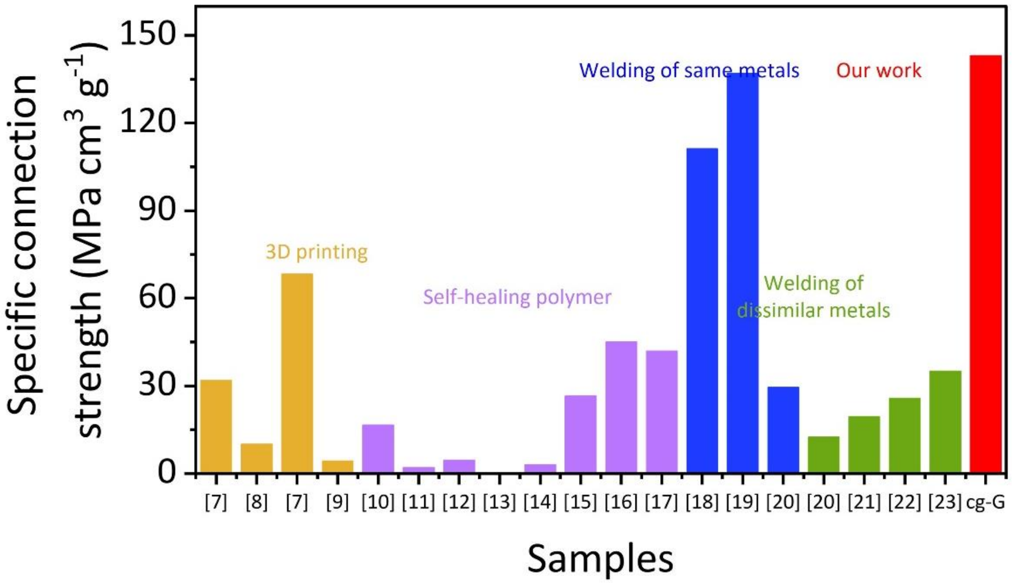

Supplementary Figure 28. Specific connection strength of *cg-G* with mortise and tenon structure, as well as various 3D printing structures, self-healing polymers, and some typical welded same and dissimilar metals.

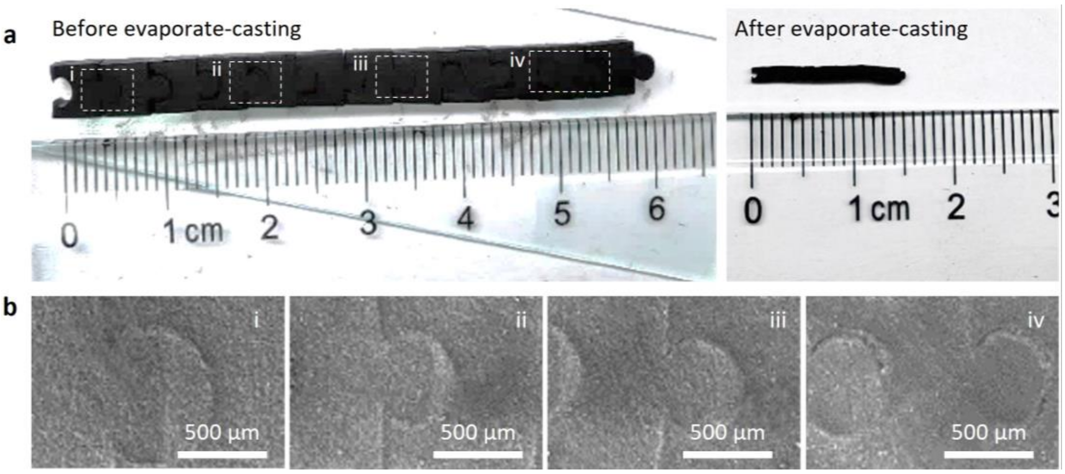

Supplementary Figure 29. Integration of *cg-G*s with mechanical interlocking structure. a, Digital images of large-size mortise and tenon joint structure combined with twelve *cg*-*G*s before and after water evaporation. **b**, SEM images of the connected parts of the mortise and tenon joint structure in **a** (i-iv) after evaporate-casting.

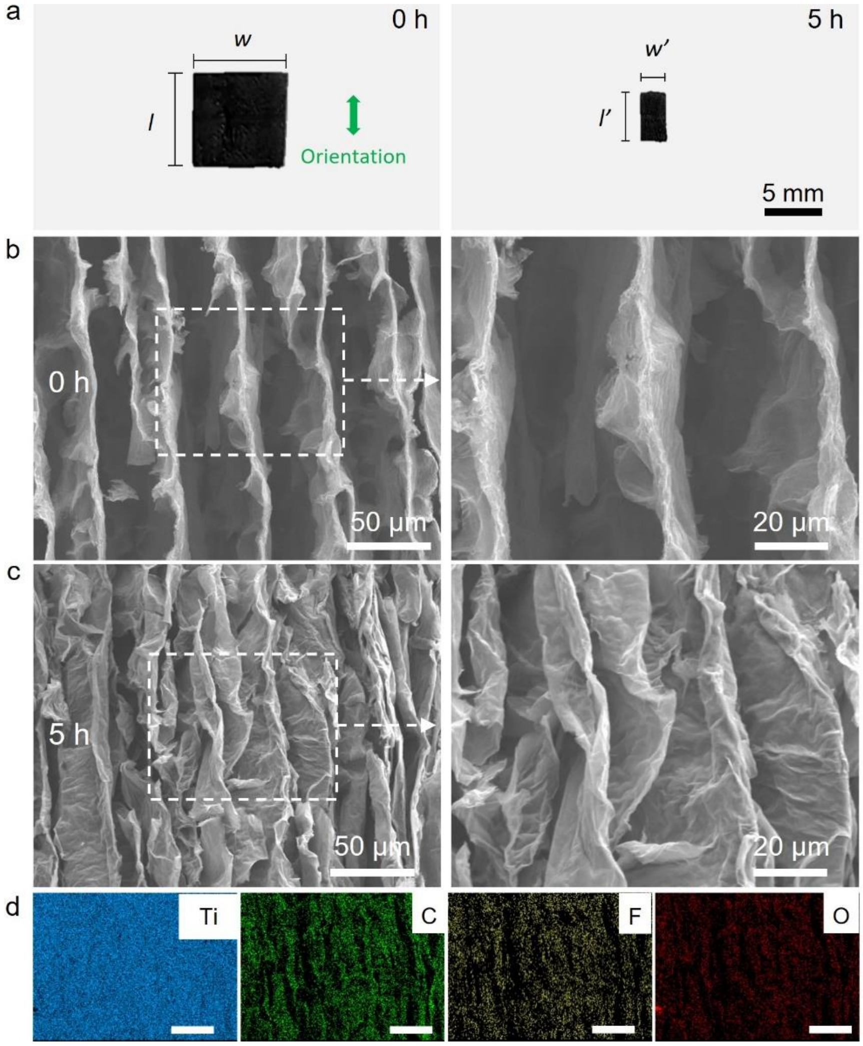

Supplementary Figure 30. Exploration into the universality of the evaporating-casting method. a, Optical images of vertically oriented MXene hydrogel with evaporation time ranging from 0 to 5 h. SEM images of the MXene hydrogel (**b**) before and (**c**) after water evaporation. **d**, EDS mappings of Ti, C, F, and O elements. Scale bar: 50 μm.

Universality plays a pivotal role in material preparation, greatly amplifying the practical applicability of a method and contributing significantly to advancements in the field of material preparation. In this work, we opted to investigate the evaporation behavior of MXene hydrogels with oriented structures. Specifically, the preparation of MXene hydrogels followed a meticulously outlined procedure. The $Ti_3C_2T_x$ dispersion (60 mg g^{-1}) was carefully poured into PTFE molds, positioned atop a 0.5 cm thick stainless-steel block in contact with liquid nitrogen. Following the complete freezing of

the $Ti_3C_2T_x$ dispersion, the resultant frozen sample was extracted from the PTFE mold and stored in a refrigerator (T = −20 °C) for a duration of 4 hours. Subsequently, the frozen gel underwent a thawing process in 5 M HCl solution at room temperature, with gentle agitation, spanning a timeframe of 6 hours. The solution was then subjected to multiple replacements with deionized water until achieving neutrality. The subsequent dialysis process culminated in the preparation of MXene hydrogel characterized by a long-range oriented microstructure.

Fig. S30 illustrates the ensuing steps wherein the obtained MXene hydrogel was meticulously sliced into dimensions of 8 mm × 8 mm × 2 mm and positioned on a PTFE substrate, allowing for spontaneous evaporation of moisture under ambient conditions. Observations from Fig. S30a elucidate a significant reduction in dimensions perpendicular to the layer direction as moisture evaporated, indicating a marked decrease in gap size, whereas changes along the layer direction remained relatively minor. After 5 hours, the complete evaporation of moisture within the MXene hydrogel culminated in its final evaporative shaping. Analogous to curvature gradient graphene hydrogels, the resultant MXene assembly manifested a regular rectangular structure, underscoring the controllable nature of MXene hydrogel shrinkage.

Further analysis through SEM images (Fig. S30b) revealed a distinct long-range ordered structure within the MXene hydrogel prior to shrinkage, characterized by an interlayer spacing of approximately 50 μm, surpassing that of *cg-G*'s 20 μm. With ongoing water evaporation, the MXene hydrogel layers exhibited wrinkling, leading to the convergence of adjacent layers and a subsequent decrease in interlayer spacing until its eventual disappearance. This behavior mirrors the shrinkage exhibited by curvature gradient graphene hydrogels. Nonetheless, the degree of wrinkling observed in MXene hydrogel layers was comparatively lower, with minimal observable changes in the shrunken MXene layers. Consequently, the degree of shrinkage along the layer direction within MXene hydrogel was diminished, with the l'/w' ratio after deformation reaching as high as 2, significantly surpassing that of curvature gradient graphene superstructures.

These findings underscore the evaporative-induced deformation behavior shared between MXene hydrogels and curvature gradient graphene hydrogels, thereby validating the proposed method’s universality. Similarly, by replicating analogous oriented or curvature gradient structures, it is hypothesized that other materials such as graphene/MXene composite hydrogels or polymer composite hydrogels can likewise exhibit comparable evaporative deformation behavior.

Supplementary Table

Supplementary Table 1. Joint parameters of *cg-G* with mortise and tenon structure, as well as various materials with combine structures, such as 3D printing structures, self-healing polymers, and some typical welded same and dissimilar metals.

Methods	Materials	Temperature (K)	Power density (W m^{-2})	Energy utilization (m^2 W^{-1})	Tensile strength (MPa)	Density (g cm^{-3})	Specific strength (Mpa cm^3 g^{-1})	Ref.
3D printing	Polypropylene-like	473.15	7479.0	1.30E-04	38	1.2	31.9	1
	Resin-carbon source composites	298.15	58.4	1.70E-02	10	~1.0	10.0	2
	Polyaryletherketone	573.15	13445.1	7.40E-05	90	1.3	68.2	1
	Elastomer resin	343.15	1715.6	5.80E-04	4.2	~1.0	4.2	3
Self-healing polymer	Epoxy/functional GO	393.15	1800.2	5.60E-04	16.5	~1.0	16.5	4
	ACON	323.15	432.0	2.30E-03	2	~1.0	2.0	5
	BCP	333.15	604.8	1.70E-03	4.4	~1.0	4.4	6
	Fe-triazole-PDMS	333.15	604.8	1.70E-03	0.2	~1.0	0.2	7
	PMMA, PA-amide	298.15	27.3	3.70E-02	3.0	~1.0	3.0	8
	Crosslinked PU	373.15	1368.7	7.30E-04	26.5	~1.0	26.5	9
	$TUEG_3$	413.15	2269.9	4.40E-04	45.0	~1.0	45.0	10
Welding of same metals	AA2024	453.15	31177.3	3.20E-05	113	2.7	41.9	11
	AA5182	673.15	76118.9	1.30E-05	300	2.7	111.1	12
	Titanium	1923.15	179728.7	5.60E-06	-	-	137.0	13
Welding of dissimilar metals	Q235/Zn12Al/6061Al	793.15	54122.1	1.80E-05	154.3	5.3	29.4	14
	Q235/Al7Si20Cu/SUS 304	893.15	66860.2	1.50E-05	65	5.3	12.4	14
	Q235/Zn15Al0.05La/ 6061 Al	693.15	42244.4	2.40E-05	102	5.3	19.4	15
	Ti6Al4V Ti alloy/2024-T6 Al alloy	923.15	17588.1	5.70E-05	158	6.2	25.7	16
	TC4 Ti alloy/316 SS	973.15	20278.7	4.90E-05	215	6.2	35.0	17
**This work**	***cg-G***	**298.15**	**10.8**	**9.20E-02**	**189.4**	**~1.4**	**135.3**	
		**308.15**	**168.3**	**5.90E-03**	**193.5**		**138.2**	
		**318.15**	**331.6**	**3.00E-03**	**196.0**		**140.0**	
		**328.15**	**501.1**	**2.00E-03**	**199.1**		**142.2**	
		**338.15**	**677.3**	**1.50E-03**	**200.0**		**143.0**	

GO: graphene oxide; ACON: secondary amide-containing cyclooctene (CO) network via carbodiimide coupling with *N*-acetylglycine; PMMA-PA amide: a hard polymethylmethacrylate (PMMA) and soft polyacrylate-amide (PA-amide) brushes that exhibit thermoplastic elastomer properties; Fe-Hpdca-PDMS-Fe-2,6-pyridinedicarboxamide (pdca) coordination complex with PDMS; crosslinked PU: the amount of the synchronous (C-ON) bond involved in fission/radical recombination that enables interrelated reprogramming, intrinsic self-healing of wider crack and recycling of the crosslinked PU; $TUEG_3$: poly(ether-thioureas) with triethylene glycol; BCP: block copolymers (PA-amide)-b-PMMA-b-(PA-amide).

Energy utilization is determined by the reciprocal of power density in material preparation, which is obtained through the computed of thermal transfer power during the bonding process. In this process, the operating temperature plays a critical role. The results indicate that our material, capable of self-tightening at both room temperature and higher temperatures, exhibits connection strength comparable to welded metals and showcases a remarkably high level of energy utilization (higher by 1 - 4 orders of magnitude than other materials).

In this context, the thermal transfer power during the material preparation process is primary composed of the combined effects of heat convection and radiation. It's worth emphasizing that this heat transfer power is a calculated value under ideal circumstances, signifying that it reflects the minimum energy necessary for the reaction to occur. This can be employed as a rough estimate for comparing the energy consumption in the preparation processes of various material systems.

Heat convective power can be calculated *via* the following formula:

$$P_c = Q/A = h \cdot \triangle T \tag{5}$$

where P_c represents heat convective power (W/m^2); Q represents the heat transferred through convection per unit of time (W); A represents the heat transfer surface area (m^2); h is the convective heat transfer coefficient (W/(m^2 °C)); $\triangle T$ is the temperature difference, typically between the surface temperature of the object and the fluid temperature (K).

Heat radiation power can be calculated *via* using the Stefan-Boltzmann Law:

$$P_r = Q/A = \sigma \cdot (T_s^4 - T_0^4) \tag{6}$$

where P_r represents heat radiation power (W/m^2); Q represents the heat radiation per unit of time (W); σ is the Stefan-Boltzmann constant; A is the radiating surface area (m^2); T_s is the surface temperature of the object (K); T_0 is the temperature of the surrounding environment (K).

References

1. J. R. C. Dizon, A. H. Espera, Jr., Q. Chen, R. C. Advincula, Mechanical characterization of 3D-printed polymers. *Addi. Manuf.* **20**, 44-67 (2018).
2. Q. Mu *et al.*, Digital light processing 3D printing of conductive complex structures. *Addi. Manuf.* **18**, 74-83 (2017).
3. D. K. Patel *et al.*, Highly stretchable and UV curable elastomers for digital light processing based 3D printing. *Adv. Mater.* **29**, 1606000 (2017).
4. A. Kausar, I. Ahmad, M. Maaza, P. Bocchetta, Self-healing nanocomposites-advancements and aerospace applications. *J. Compos. Sci* **7**, 148 (2023).
5. J. A. Neal, D. Mozhdehi, Z. Guan, Enhancing mechanical performance of a covalent self-healing material by sacrificial noncovalent bonds. *J. Am. Chem. Soc.* **137**, 4846-4850 (2015).
6. D. Borah *et al.*, Directed self-assembly of PS-b-PMMA block copolymer using HSQ lines for translational alignment. *J. Mater. Chem. C* **1**, 1192-1196 (2013).
7. X. Y. Jia, J. F. Mei, J. C. Lai, C. H. Li, X. Z. You, A highly stretchable polymer that can be thermally healed at mild temperature. *Macromol. Rapid Commun.* **37**, 952-956 (2016).
8. Y. Chen, Z. Guan, Self-healing thermoplastic elastomer brush copolymers having a glassy polymethylmethacrylate backbone and rubbery polyacrylate-amide brushes. *Polymer* **69**, 249-254 (2015).
9. L. F. Fan, M. Z. Rong, M. Q. Zhang, X. D. Chen, Dynamic reversible bonds enable external stress-free two-way shape memory effect of a polymer network and the interrelated intrinsic self-healability of wider crack and recyclability. *J. Mater. Chem. A* **6**, 16053-16063 (2018).
10. Y. Yanagisawa, Y. Nan, K. Okuro, T. Aida, Mechanically robust, readily repairable polymers *via* tailored noncovalent cross-linking. *Science* **359**, 72-76 (2018).
11. F. Balle *et al.*, Influence of heat treatments on the mechanical properties of ultrasonic welded AA 2024/CF-PA66-joints. *Adv. Eng. Mater.* **15**, 837-845 (2013).

12. M. Paidar, A. Asgari, O. O. Ojo, A. Saberi, Mechanical properties and wear behavior of AA5182/WC nanocomposite fabricated by friction stir welding at different tool traverse speeds. *J. Mater. Eng. Perform.* **27**, 1714-1724 (2018).
13. D. H. Won, T. S. Bae, S. Ohkawa, F. Watari, The influence of output current on the tensile strength of laser-welded titanium joints. *Met. Mater. Int.* **9**, 493-496 (2003).
14. W. Lu, Y. Zhang, D. Yu, D. Sun, H. Li, Research progress on control strategy of intermetallic compounds in welding process of heterogeneous materials. *steel research int.* **93**, 2100427 (2022).
15. R. Weifeng, Study on brazing of aluminum/steel dissimilar metals, Dalian Jiaotong University, 2016.
16. Z. Ma, C. Wang, H. Yu, J. Yan, H. Shen, The microstructure and mechanical properties of fluxless gas tungsten arc welding-brazing joints made between titanium and aluminum alloys. *Mater. Des.* **45**, 72-79 (2013).
17. J. Li *et al.*, Benefits of interfacial regulation with interlayers in laser welding Ti_6Al_4V/316L steel. *Opt. Laser Technol.* **125**, 106007 (2020).
