# Supplementary material for: Evaporate-casting of curvature gradient graphene superstructures for ultra-high strength structural materials
Source: Nat Commun. 2024 Jul 14;15:5917. doi: 10.1038/s41467-024-50191-6 (PMC11247093; doi:10.1038/s41467-024-50191-6)
Supplement: Supplementary file 3 — Description of Additional Supplementary Files [file 41467_2024_50191_MOESM3_ESM.pdf]

Description of Additional Supplementary Files

File Name: Supplementary Movie 1

Description: The deformation processes of cross-shaped *cg-Gs* with different axis-center distances.

File Name: Supplementary Movie 2

Description: Mechanical performance display of clasped structure. A single interlocking structure could pull up a weight (1 kg)
